# Cbp1, a fungal virulence factor under positive selection, forms an effector complex that drives macrophage lysis

**DOI:** 10.1371/journal.ppat.1010417

**Published:** 2022-06-22

**Authors:** Dinara Azimova, Nadia Herrera, Lucian Duvenage, Mark Voorhies, Rosa A. Rodriguez, Bevin C. English, Jennifer C. Hoving, Oren Rosenberg, Anita Sil

**Affiliations:** 1 University of California San Francisco, San Francisco, California, United States of America; 2 AFRICA Medical Mycology Research Unit, Institute of Infectious Diseases and Molecular Medicine, University of Cape Town, Cape Town, South Africa; 3 University of California Davis, Davis, California, United States of America; Tulane University School of Medicine, UNITED STATES

## Abstract

Intracellular pathogens secrete effectors to manipulate their host cells. *Histoplasma capsulatum* (*Hc*) is a fungal intracellular pathogen of humans that grows in a yeast form in the host. *Hc* yeasts are phagocytosed by macrophages, where fungal intracellular replication precedes macrophage lysis. The most abundant virulence factor secreted by *Hc* yeast cells is Calcium Binding Protein 1 (Cbp1), which is absolutely required for macrophage lysis. Here we take an evolutionary, structural, and cell biological approach to understand Cbp1 function. We find that Cbp1 is present only in the genomes of closely related dimorphic fungal species of the Ajellomycetaceae family that lead primarily intracellular lifestyles in their mammalian hosts (*Histoplasma*, *Paracoccidioides*, and *Emergomyces*), but not conserved in the extracellular fungal pathogen *Blastomyces dermatitidis*. We observe a high rate of fixation of non-synonymous substitutions in the Cbp1 coding sequences, indicating that Cbp1 is under positive selection. We determine the *de novo* structures of *H*c H88 Cbp1 and the *Paracoccidioides americana* (Pb03) Cbp1, revealing a novel “binocular” fold consisting of a helical dimer arrangement wherein two helices from each monomer contribute to a four-helix bundle. In contrast to Pb03 Cbp1, we show that *Emergomyces* Cbp1 orthologs are unable to stimulate macrophage lysis when expressed in the *Hc cbp1* mutant. Consistent with this result, we find that wild-type *Emergomyces africanus* yeast are able to grow within primary macrophages but are incapable of lysing them. Finally, we use subcellular fractionation of infected macrophages and indirect immunofluorescence to show that Cbp1 localizes to the macrophage cytosol during *Hc* infection, making this the first instance of a phagosomal human fungal pathogen directing an effector into the cytosol of the host cell. We additionally show that Cbp1 forms a complex with Yps-3, another known *Hc* virulence factor that accesses the cytosol. Taken together, these data imply that Cbp1 is a fungal virulence factor under positive selection that localizes to the cytosol to trigger host cell lysis.

## Author summary

The members of the Ajellomycetaceae fungal family are human pathogens that are responsible for a rising number of mycoses around the world. Calcium binding protein 1 (Cbp1) is a virulence factor that is present in the genomes of the Ajellomycetaceae species that lead primarily intracellular lifestyles, including *Histoplasma*, *Paracoccidioides*, and *Emergomyces* but not *Blastomyces*, which remains largely extracellular during infection. Both *Paracoccidioides* and *Histoplasma* Cbp1 homologs are able to cause lysis of macrophages whereas *Emergomyces* homologs cannot. This result is consistent with *Emergomyces africanus* natural infection of macrophages, during which the yeast cells can replicate but cannot actively lyse the host cell. Despite divergence of the primary sequence of *Histoplasma* and *Paracoccidioides* Cbp1 homologs, their protein structures are remarkably similar and reveal a novel fold. During infection, Cbp1 enters the cytosol of the host macrophage, making it the first known virulence factor from an intracellular human fungal pathogen that localizes to the cytosol of the host cell. We also show that Cbp1 forms a complex with another cytosolic virulence factor, Yps-3. Taken together, these studies significantly advance our understanding of *Histoplasma* virulence.

## Introduction

Intracellular pathogens contend with the need to co-opt their host cells to fashion a replicative niche and the requirement to manipulate host-cell survival, sometimes destroying their host cell at the appropriate time to promote exit and further dissemination. To shape their host environment, intracellular pathogens secrete a number of diverse molecules to help the pathogen evade detection by the immune system, scavenge nutrients, and assist in replication and dissemination throughout their host. Bacteria, viruses, parasites, and fungi all secrete effectors, many of which are proteins that are capable of manipulating the biology of the host cell [1–5]. Pathogens that reside in nutritionally limiting phagosomal compartments that are normally slated for degradation must be particularly adept at remodeling this niche to make it compatible with microbial replication [6–8]. In the case of human pathogenic fungi, we understand much less about secreted effectors of virulence [9–11] compared to bacterial or parasite pathogens. Here we explore the biology of a key virulence effector from the intracellular human fungal pathogen *Histoplasma capsulatum (Hc*).

*Hc* is the causative agent of histoplasmosis and can cause disease even in immunocompetent humans [11–14]. This fungus is found world-wide, and in the Central and Eastern United States, *Histoplasma* species are commonly found in the soil of the Ohio and Mississippi River Valleys [15–17]. *Hc* is a thermally dimorphic fungus that grows in the soil in a multicellular filamentous form that produces conidia [18–20]. These filamentous fragments and conidia can be inhaled by a mammalian host, where host temperature induces a transition to a pathogenic yeast form. *Hc* yeast are phagocytosed by resident alveolar macrophages in the lungs, where they replicate within a modified phagosomal compartment [21–25]. Once *Hc* replicates to high levels, the host cells lyse, thereby releasing yeast cells and facilitating further spread of the fungus to neighboring macrophages.

One of the most abundant yeast-phase proteins secreted by *Hc* into culture supernatants is a small 7.8 kDa protein known as Calcium binding protein 1 (Cbp1) [26–29]. As determined previously by an NMR structure and other biochemical analyses, Cbp1 is a dimer with three intramolecular disulfide bridges that make it highly stable [30, 31]. The mature secreted form of Cbp1 is 78 amino acids in length, has no known domains, and, prior to this work, had only one known homolog in the closely related fungus *Paracoccidioides*. Interestingly, Cbp1 is a critical virulence factor that is required for virulence of *Hc* in both macrophage and mouse models of infection [29, 32, 33]. *Hc* that lacks Cbp1 is able to grow to high levels within the macrophage but is unable to lyse either primary murine bone marrow derived macrophages (BMDMs) or primary alveolar macrophages [32, 33]. These data suggest that Cbp1 is a protein effector used by *Hc* to induce lysis of macrophages. Recent work from our lab has demonstrated that Cbp1 actively triggers apoptosis of macrophages through the integrated stress response (ISR), a cascade that is triggered in the cytosol of infected macrophages. The secretion of a functional Cbp1 is absolutely required for this stress response to be initiated in infected macrophages [32]. Additionally, we have previously shown that the *Paracoccidioides americana* (Pb03) homolog of Cbp1 is capable of fully restoring lytic capability when expressed in a *Hc cbp1* mutant background [32], suggesting that *Hc* is not the only species that could be using Cbp1 to elicit host cell death. How Cbp1 triggers the ISR and macrophage death is still not fully understood.

Another known secreted effector of *Hc* is the Yeast phase specific 3 (Yps-3) protein [34]. Like Cbp1, Yps-3 is a secreted factor that is only produced by the pathogenic yeast phase of the fungus and has been previously shown to be important for virulence in a murine model of histoplasmosis using an RNA interference strain with reduced expression of *YPS3* [34]. Yps-3 is thought to coat the surface of the yeast cell by binding to chitin [34]. Neither Cbp1 nor Yps-3 has been shown to interact with any other fungal proteins.

In this study, we used evolutionary, structural, and cellular approaches to study *Hc* Cbp1 and its homologs. We found new, previously unannotated Cbp1 homologs in the genomes of *Paracoccidioides* and *Emergomyces* species. *Emergomyces* are newly emerging human fungal pathogens that are responsible for a rising incidence of mycoses in immunocompromised humans worldwide [35–38]. *Emergomyces* species include *Emmonsia crescens*, *(E*. *crescens*) and *Emergomyces africanus*, *orientalis*, *and pasteurianus (Es*. *africanus*, *Es*. *orientalis*, and *Es*. *pasteurianus*). In contrast, despite its close evolutionary relationship to *Hc*, the extracellular pathogen *Blastomyces* does not harbor a Cbp1 homolog. We determined a new structure of both *Hc* and *Pb* homologs of Cbp1, thereby revealing a novel protein fold. To perform a functional analysis of Cbp1 homologs, we expressed Cbp1 from Pb03 and *Emergomyces* species in the *Hc cbp1* mutant and assessed the ability of each homolog to complement the host lysis defect. Despite the conservation of Cbp1 homologs in the genomes of *Emergomyces* species, these proteins were unlike the Pb03 Cbp1 in that they could not restore lysis during *Hc* infection. The interaction between BMDMs and wild-type *Es*. *africanus* had not previously been examined; we discovered that this fungus is capable of robust intracellular replication within BMDMs without any evidence of host-cell lysis, consistent with the inability of *Emergomyces* Cbp1 to complement the *Hc* mutant. Finally, to uncover the site of action of *Hc* Cbp1, we determined that it enters the macrophage cytosol from the *Hc*-containing phagosome. Biochemical analyses revealed that Cbp1 forms a complex with Yps-3 and, coupled with our observation that *yps3Δ* mutants cannot achieve maximal lysis of macrophages, suggested the formation of a cytosolic effector complex that mediates host-cell death. Given that Cbp1 1) is unique to the Ajellomycetaceae, 2) shows significant non-synonymous relative to synonymous sequence divergence among homologs, and 3) mediates differential lysis between intracellular pathogens, we conclude that the virulence factor Cbp1 is a rapidly evolving protein that is critical for macrophage manipulation by *Hc*.

## Results

### Cbp1 is conserved amongst the Ajellomycetaceae with the exception of *Blastomyces* species

Cbp1 homologs are not broadly present in the fungal kingdom [32]. Due to its critical role during intracellular infection, we hypothesized that Cbp1-dependent virulence strategies could be conserved amongst closely related thermally dimorphic fungal species in the Ajellomycetaceae family that are human intracellular pathogens. To identify all putative Cbp1 homologs, we expanded the Cbp1 alignment previously published in [26, 32] by building a Hidden Markov Model (HMM) to search through the annotated protein sets generated from all the additional published genomes of *Emergomyces*, *Blastomyces*, and *Paracoccidioides* species deposited in GenBank. We detected five *Emergomyces* and two *Paracoccidioides* Cbp1 homologs. To identify the *Emergomyces* homologs, we first defined a syntenic region based on the previously established *Hc* and *Pb* homologs. When we interrogated the corresponding syntenic region *in Emergomyces* species, we uncovered three new *Emergomyces* homologs. All three share the conserved two intron structure found in the *Hc* and *Pb CBP1* genes [32]. In the case of *Es*. *pasteuriana*, we discovered two Cbp1 homologs (*Es*. *pasteuriana_1* and *Es*. *pasteuriana_2)*. One homolog was in the syntenic region and maintained the two-intron structure whereas the other was located in a different genomic region and had a three-intron gene structure. Using the *Es*. *pasteuriana* homologs as templates, we searched through the unannotated *Es*. *orientalis* genome and found one additional syntenic homolog (*Es*. *orientalis_1*). A potential non-syntenic *Es*. *orientalis* homolog was not studied further since it contained two in-frame stop codons and was challenging to predict due to lack of a canonical splice site. In contrast, we found that Cbp1 was not present in any of the *Blastomyces* genomes. Unlike the other three major members of this fungal family, *Blastomyces* leads a largely extracellular lifestyle *in vivo* [35, 39, 40], suggesting that Cbp1 could be a virulence factor that represents a specialized adaptation to an intracellular lifestyle inside of a mammalian host.

**Fig 1:**
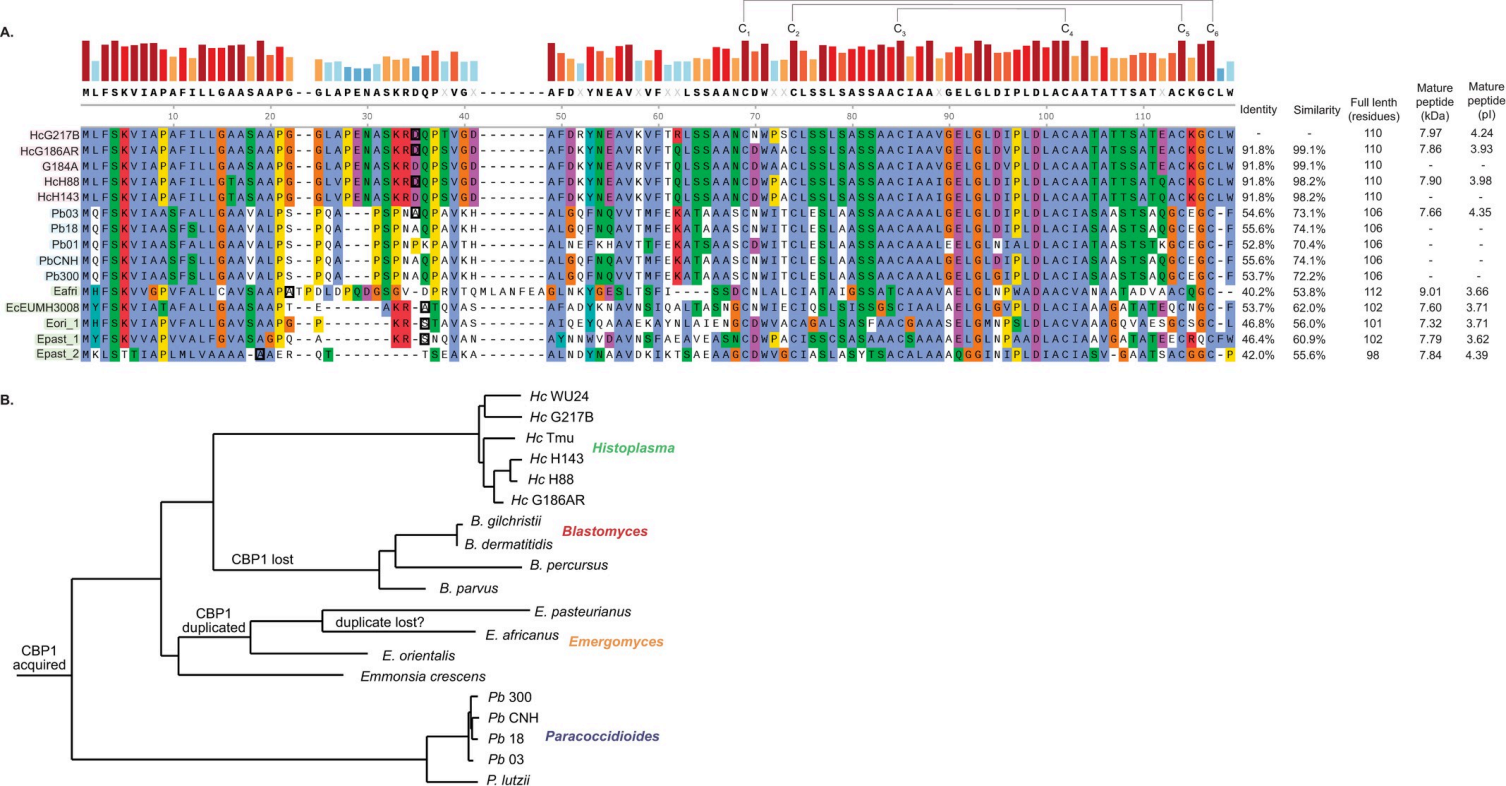
Cbp1 is conserved among closely related thermally dimorphic fungi that are intracellular pathogens of humans. A. Protein alignments of *Hc* G217B Cbp1 and closely related homologs. The sequences of the *Histoplasma* homologs (G217B, G186AR, G184A, H88, and H143) highlighted in green, *Paracoccidioides* homologs (Pb03, Pb18, Pb01, PbCNH, and Pb300) highlighted in blue, and the *Emergomyces* and *Emmonsia* homologs (*Es*. *Africanus*, *E*. *crescens*, *Es*. *orientalis*, *Es*. *pasteurianus_1*, *and Es*. *pasteurianus_2*) highlighted in yellow are shown. The start of the mature peptide as determined by mass spectrometry of the *Hc* G217B, *Hc* G186AR, *Hc* H88, Pb03, *Es*. *africanus*, *E*. *crescens*, *Es*. *orientalis*, *Es*. *pasteurianus_1*, and *Es*. *pasteurianus_2* homologs is indicated with a black box for each. The six cysteine residues that form intramolecular disulfide bonds are indicated, as well as the percent identity and similarity to the *Hc* G217B, the full length of the immature protein, and the molecular weight and pI of the mature peptides. B. Species tree that tracks the acquisition and loss events of Cbp1 in Ajellomycetaceae species that are human fungal pathogens.

We aligned the new Cbp1 homologs to the Cbp1 consensus sequence generated by the HMM (Fig 1A) along with the previously characterized *Hc* and *Pb03* Cbp1 homologs, which have been previously shown to be necessary for macrophage lysis [29, 32, 33]. The 5’ portion of the Cbp1 coding sequence, which contains the putative signal peptide necessary for extracellular secretion of the protein, is well conserved amongst all the homologs. However, the residues flanking the putative cleavage site of the signal sequence, as based upon *Hc* G217B Cbp1, are poorly conserved. The N-terminus of the mature protein is fairly well conserved whereas the latter half of the predicted coding sequence is highly conserved, especially the six cysteines that form the three intramolecular disulfide bridges [30, 31].

When comparing the sequences of the Cbp1 homologs on a primary amino acid level relative to the sequence of G217B Cbp1, it is clear that these sequences diverge rapidly. We see that the Cbp1 sequences within each genus (*Histoplasma*, *Paracoccidioides*, and *Emergomyces*) are much closer to each other than they are to homologs from a different genus. The percent identity between G217B and G186AR/H88 Cbp1 is high, at 91.8% (Fig 1A). The next closest sequence to G217B is that of Pb03 and other *Paracoccidioides* species, which range between 52–55% identical (Fig 1A). *Emergomyces* Cbp1 homologs show less identity to G217B Cbp1, ranging between 38–53% (Fig 1A). In order to distinguish whether the evolutionary divergence of Cbp1 protein sequences is due to natural selection rather than genetic drift of unconstrained residues, we considered the rate of non-synonymous (dN) relative to synonymous (dS) codon changes. Specifically, we used the PAML program [41] to fit models of positive selection and neutral drift to a codon-level alignment of the 11 Cbp1 orthologs syntenic to G217B *Hc* Cbp1. Based on likelihood ratio tests, positive selection is significantly more likely than neutral drift both when modeling dN/dS of neutral positions as a beta distribution (M8/M7 test, p = 7.9x10^-6^) as well as when using the stricter parameterization of a single dN/dS value for neutral positions (M2a/M1a test, p = 4.0x10^-5^) [41].

The most parsimonious explanation for the emergence of Cbp1 in this fungal family is that Cbp1 evolved in an early common ancestor and was then subsequently lost from the *Blastomyces* ancestral species as it diverged away from the other members of Ajellomycetaceae (Fig 1B). Similarly, the duplication of the Cbp1 gene in *Es*. *pasteuriana* and *Es*. *orientalis* may have occurred in a common ancestor of the *Es*. *orientalis*, *Es*. *pasteuriana*, and *Es*. *africanus* species. We note that this implies either subsequent loss of the Cbp1 paralog in *Es*. *africanus* or omission of this gene from the *Es*. *africanus* assembly (GCA_001660665.1), which is highly fragmented and 3MB smaller than the other *Emergomyces* genomes. Taken together, these data suggest that Cbp1 was gained early in the evolution of the Ajellomycetaceae and diverged rapidly among the various species in this family due, at least in part, to positive selection by factors that cannot be inferred from sequence analysis alone.

### Structures of Hc H88 and Pb03 Cbp1 reveal a novel helical “binocular” fold

**Fig 2:**
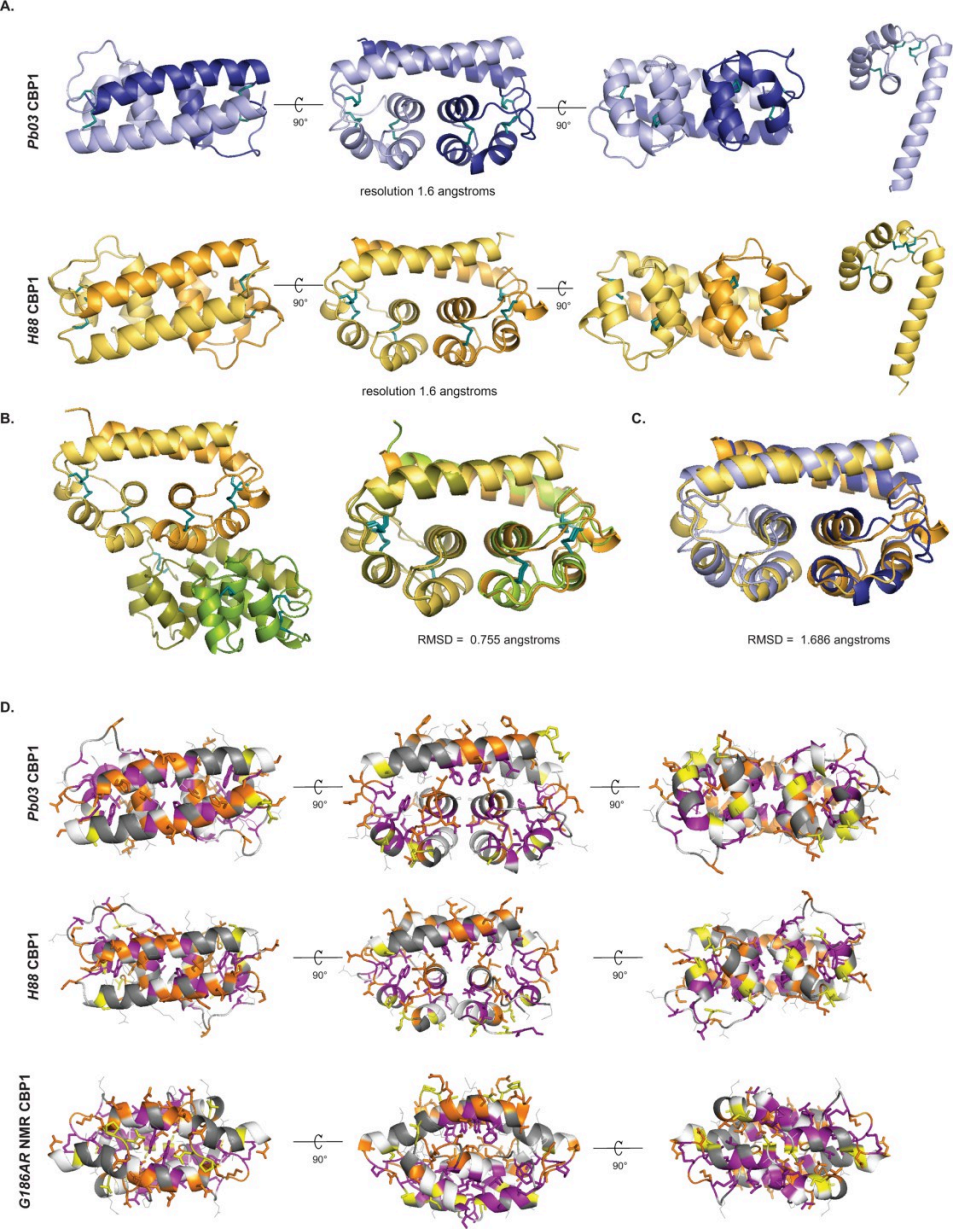
Crystal structures of H88 and Pb03 Cbp1 reveal a novel “binocular” fold. A. Structures of *Pb03* and *H88* Cbp1 as determined by crystallographic methods are shown in native dimer form and as a monomer. B. Asymmetric unit of H88 Cbp1 shows a dimer of dimers that interact though the C-terminal helical bundles. The two dimers in the H88 asymmetric unit are aligned and the RMSD score was determined to be 0.755 angstroms. C. Structural alignments of *Pb03* and *H88* Cbp1 structures reveal a highly similar fold with an RMSD value of 1.686 angstroms. D. Prior experiments in our laboratory determined that the purple residues are required for secretion, the orange residues are absolutely required for lysis, and the yellow residues contribute to maximal lysis [32]. When these residues are modeled on the *Pb03* or *H88* crystal structure (top two rows), the purple residues face inwards and the orange and yellow residues are predicted to be on the surface of the protein facing outwards. In contrast, modeling the purple, orange, and yellow residues on the NMR structure does not result in a consistent pattern.

**Fig 3:**
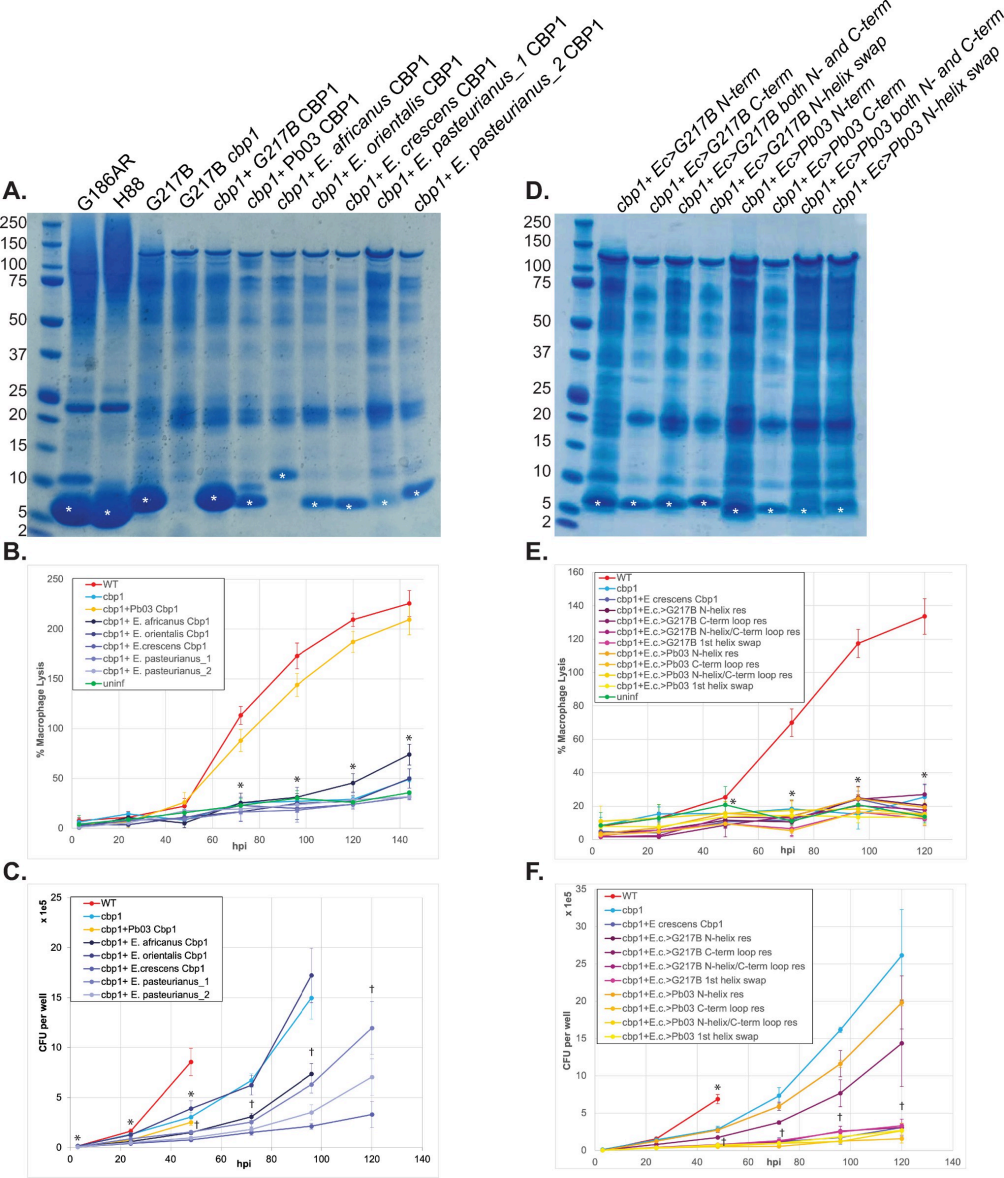
*Emergomyces* species contain Cbp1 homologs in their genomes that can be expressed and secreted by the *Hc* G217B strain but are insufficient to confer macrophage lysis. A. InstantBlue stained SDS-PAGE gel of culture supernatants of *Hc* G186AR, *Hc* H88, *Hc* G217B, *Hc* G217B *cbp1* mutant, and *Hc* G217B *cbp1* mutant expressing the G217B, *P*. *americana (Pb03)*, *E*. *crescens*, *Es*. *orientalis*, *Es*. *pasteurianus_1*, *Es*. *pasteurianus_2*, *or Es*. *africanus* Cbp1. White asterisk denotes the bands that were excised for mass spectrometric analysis to confirm their identity as the appropriate Cbp1. Molecular weight standards (kD) are marked on the left. B. *Hc cbp1* mutant isolates expressing the *Emergomyces* Cbp1 homologs were used to infect BMDMs at an MOI of 1 as described in the Materials and Methods. “WT” indicates the *Hc* G217B *ura5* mutant carrying the *URA5* gene on a plasmid. LDH release was used to quantify percent host cell lysis. C. Intracellular growth of *Hc cbp1* mutant isolates expressing the *Emergomyces* Cbp1 homologs was determined from BMDM infection as described in the Materials and Methods. “WT” indicates the *Hc* G217B *ura5* mutant carrying the *URA5* gene on a plasmid. At 48 hpi, CFU counts were terminated for the WT *Hc* infection because the extent of host-cell lysis made it difficult to distinguish between intracellular and extracellular growth of *Hc*. D. InstantBlue stained SDS-PAGE gel of culture supernatants of G217B *Hc cbp1* mutant expressing the chimeric constructs that convert key residues from *E*. *crescens* Cbp1 into their counterpart residue from G217B or Pb03 Cbp1 in the N-terminus, the C-terminal loops, both the N-terminus and C-terminal loops, or a 1^st^ helix swap. White asterisk denotes the bands that were excised for mass spectrometric analysis to confirm their identity as the appropriate chimeric Cbp1. E. *Hc cbp1*-mutant isolates expressing *E*. *crescens* chimeric Cbp1 homologs were used to infect BMDMs and LDH release was quantified. F. Intracellular growth of *Hc cbp1* mutant isolates expressing the *E*. *crescens* chimeric Cbp1 homologs was determined from BMDM infection as described in the Materials and Methods. “WT” indicates the *Hc* G217B *ura5* mutant carrying the *URA5* gene on a plasmid. At 48 hpi, CFU counts were terminated for the WT *Hc* infection because the extent of host-cell lysis made it difficult to distinguish between intracellular and extracellular growth of *Hc*. For LDH analyses, asterisk indicated p-value <0.05 relative to WT according to a t-test. For CFU analyses, asterisk indicates p-value <0.05 according to t-test relative to WT whereas dagger indicates p<0.05 relative to the cbp1 mutant.

To determine if there is any structural similarity between these homologs and the previously published G186AR NMR Cbp1 structure [30], we used X-ray crystallography to solve the structure of a subset of homologs. Since Cbp1 makes up the vast majority of the *Hc* secretome [42], we purified the protein directly from culture supernatants with minimal perturbation of the native protein. We purified a range of Cbp1 homologs. *Hc* G217B, *Hc* G186AR, and *Hc* H88 Cbp1s were purified from their respective *Histoplasma* strains whereas Pb03, *E*. *crescens*, *and Es*. *africanus* Cbp1 homologs were expressed in *Hc* (S1 Fig) as described in Materials and Methods. Mass spectrometric analysis of *Pb03* Cbp1 revealed two signal sequence cleavage sites, which likely explain the doublet on SDS-PAGE (Figs 3A and S1). Sequence inspection of *Es*. *africanus* Cbp1 revealed a 7-aa insertion relative to other Cbp1s and mass spectrometry supported a more N-terminal signal peptide cleavage site, accounting for at least some of the decreased mobility on SDS-PAGE (Figs 1 and 3A and S1). Crystals of *Hc* H88 and Pb03 Cbp1 homologs formed after a week and diffracted to 1.6 Å (Fig 2A). To resolve the phases, a heavy atom derivative was prepared by soaking the Pb03 crystals in K_2_PtCl_4_. Two datasets were collected at the L III Pt-edge at 11562 eV and at a remote energy of 13500 eV. Multiple anomalous diffraction (MAD) data were used to search for the heavy atom positions using the Phaser program in the Phenix crystallography programming suite. The structure of Pb03 was truncated at C-terminal residues 65–67 from chain A and 67–68 from chain B, as the remaining amino acids were not resolved in the refined density. The final refinement statistics were Rwork/free = 0.21 / 0.22 and the data were deposited under PDBID 7R6U (Table 1). The diffraction data from *Hc* H88 was resolved by molecular replacement using the Pb03 model. Using this model in the Phaser program in the Phenix crystallography programming suite, we obtained a TFZ score of 8.4 and an LLG score of 63.271, supporting the validity of the molecular replacement solution. The final refinement statistics were Rwork/free = 0.21 / 0.23 and the data were deposited under PDBID 7R79 (Table 1).

**Table 1:** Data collection and refinement statistics.

	H88 Cbp1	Pb03 Cbp1	Pb03 Cbp1- E1	Pb03 Cbp1- E2
**Wavelength**	1.11	1.11	1.07	0.90
**Resolution range**	42.27–1.6 (1.657–1.6)	44.14–1.55 (1.605–1.55)	43.95–3.0 (3.108–3.0)	43.89–3.0 (3.107–3.0)
**Space group**	C 1 2 1	P 41 21 2	P 41 21 2	P 41 21 2
**Unit cell**	88.55 46.697 71.255 90 109.097 90	46.507 46.507 140.123 90 90 90	46.299 46.299 139.775 90 90 90	46.231 46.231 139.716 90 90 90
**Total reflections**	240007 (24131)	559121 (43919)	323980 (31184)	324834 (35562)
**Unique reflections**	36276 (3571)	23244 (2261)	3415 (334)	3408 (336)
**Multiplicity**	6.6 (6.8)	24.1 (19.4)	94.9 (93.4)	95.3 (105.8)
**Completeness (%)**	99.32 (98.29)	99.92 (99.56)	99.85 (100.00)	99.85 (100.00)
**Mean I/sigma(I)**	21.74 (1.83)	18.12 (0.50)	32.41 (6.35)	40.10 (8.52)
**Wilson B-factor**	25.49	24.85	66.00	67.64
**R-merge**	0.04273 (0.9169)	0.1218 (4.973)	0.2405 (1.501)	0.1782 (1.136)
**R-meas**	0.04643 (0.9925)	0.1244 (5.107)	0.2418 (1.509)	0.1792 (1.142)
**R-pim**	0.01792 (0.3761)	0.0253 (1.142)	0.0245 (0.1551)	0.01811 (0.11)
**CC1/2**	1 (0.758)	1 (0.219)	1 (0.972)	1 (0.989)
CC*	1 (0.929)	1 (0.6)	1 (0.993)	1 (0.997)
**Anomalous completeness**	-	-	100.0 (100.0)	100.0 (100.0)
**Anomalous multiplicity**	-	-	55.5 (51.3)	55.7 (58.3)
**DelAnom correlation**	-	-	0.934 (0.265)	0.948 (0.313)
**Mid-Slope of Anom Normal**	-	-	1.889	2.147
**Reflections used in refinement**	36270 (3571)	23226 (2251)	-	-
**Reflections used for R-free**	1473 (145)	1129 (106)	-	-
**R-work**	0.2136 (0.3079)	0.2101 (0.3366)	-	-
**R-free**	0.2352 (0.3074)	0.2238 (0.3334)	-	-
**CC(work)**	0.951 (0.788)	0.959 (0.614)	-	-
**CC(free)**	0.944 (0.717)	0.961 (0.716)	-	-
**Number of non-hydrogen atoms**	2333	1145	-	-
**macromolecules**	2146	1048	-	-
**ligands**	0	0	-	-
**solvent**	187	97	-	-
**Protein residues**	306	152	-	-
**RMS(bonds)**	0.006	0.008	-	-
**RMS(angles)**	0.87	0.99	-	-
**Ramachandran favored (%)**	97.32	97.97	-	-
**Ramachandran allowed (%)**	2.68	2.03	-	-
**Ramachandran outliers (%)**	0.00	0.00	-	-
**Rotamer outliers (%)**	0.00	0.00	-	-
**Clash score**	2.62	0.00	-	-
**Average B-factor**	32.83	35.31	-	-
**macromolecules**	32.80	34.62	-	-
**solvent**	44.19	42.71	-	-
**PDB ID**	7R79	7R6U	-	-

Statistics for the highest-resolution shell are shown in parentheses.

The asymmetric unit for the Pb03 Cbp1 crystal contained a single dimer, whereas the asymmetric unit for the H88 crystal contained a dimer of dimers (Fig 2A and 2B). An alignment of the two dimers of H88 Cbp1 structure gave a root mean squared deviation (RMSD) value of 0.755 Å, indicating the structure remains largely unchanged amongst the dimers (Fig 2B). An alignment of the Pb03 and the *Hc* CBP1 dimers gave an RMSD of 1.6 Å, indicating the structures of the distinct homologs are conserved (Fig 2C). These structures had no significant structural homology to any known proteins according to DALI [43] or VAST [44] searches but are highly similar to each other as can be seen when the dimers of Pb03 and H88 are aligned (Fig 2C). The Cbp1 structures displayed an alpha-helical dimer arrangement with an intermolecular four-helix bundle fold at the core formed from the 3^rd^ and 4^th^ helices of each monomer, creating a binocular shape, and thus we dubbed this new arrangement as a “binocular” fold. The bundle formed by two of the three C-terminal helices of each monomer contains a number of aliphatic residues (Ile55, Pro56, Leu59, Ala63) that pack against the longer N-terminal helices (S2A Fig). The N-terminal helices from both monomers interact with each other in an anti-parallel manner and their register relative to each other appears to be determined by the N-terminal residues packing against the aliphatic patch underneath them. For both Pb03 and H88 Cbp1, the three disulfide bonds present in each monomer orient the alpha helices so that the aromatic residues (Phe12, Phe19, Trp30) are packed in the interior of the protein, creating the hydrophobic core (S2A Fig).

The Pb03 and H88 structures were similar when they were aligned, yielding an RMSD of 1.6 Å (Fig 2C). An emerging property of the Cbp1 structures is the conservation of negative charge over various surfaces of the protein at neutral pH (S2C Fig) [45, 46]. The Pb03 structure has a clear negative charge at the C-terminal end of the protein and was partially positive at the interface between the N-terminal helices. The H88 structure was not as clearly polarized but did display a groove of strong negative charge on its C-terminal side. These charges were clearly visible in both the dimers and individual monomers, indicating intrinsic properties of the monomer of the protein (S2C Fig). When the tertiary structures of *Emergomyces* homologs were modelled [47] on the Pb03 Cbp1 backbone, the variation in surface charge was evident, including models of a hypothetical heterodimer formed from *Es*. *pasteurianus_1* and _2 (S2D Fig).

The previously published *Hc* G186AR NMR Cbp1 structure [30] is distinct from the X-ray crystallography structures determined in this work. When the new structures were aligned against the G186AR NMR structure, the resulting RMSD value was 5.6 Å and 5.8 Å for Pb03 and H88 Cbp1, respectively (S2B Fig), consistent with the NMR structure not being found by DALI and VAST searches of the crystal structures. In this alignment, a major difference lies in the orientation of the three C-terminal alpha-helices in the crystal structure vs. the NMR structure. In the crystal structure, the C-terminal helices were perpendicular to the N-terminal helices, whereas in the NMR structure they are parallel. In the crystal structure, the N-terminal helices were naturally more linear, resulting in an alignment in a different register relative to each other, and suggesting alternate helix-helix interaction pairs. Finally, in the NMR structure, the two monomers are intercalated, whereas in the crystal structures this was not the case.

In prior work, we used alanine scanning to identify Cbp1 residues that are critical for function of G217B Cbp1 [32] (Fig 2D). The alanine scan identified residues that, when mutated to alanine, resulted in Cbp1 variants which were (1) incapable of being secreted, (2) capable of being secreted but only caused partial macrophage lysis, or (3) capable of being secreted but completely deficient in macrophage lysis capability. Notably, when we modeled these residues on the Pb03 or H88 crystal structure, we found that the residues that were required for Cbp1 protein secretion were all facing inwards away from the protein surface, suggesting they are important for proper folding (Fig 2D). This observation was consistent with a role in proper packing of the hydrophobic core. The majority of the residues that were necessary or contributed to Cbp1 lytic capability had side chains that were oriented out towards the external surface and were preferentially located in the N-terminal helix, suggesting that those residues create surfaces that are necessary for Cbp1 function. We did not observe concordance between the alanine mutant phenotypes and the location of the corresponding residues on the *Hc* G186AR NMR structure (Fig 2D). Thus, the alanine scan data is highly consistent with the newly determined crystal structures of *Hc* H88 Cbp1 and its homologs.

### *Emergomyces* Cbp1 homologs cannot complement the *Hc cbp1* mutant

To further probe the function of the newly identified Cbp1 homologs, we expressed them in *Hc* to determine whether they could complement the *cbp1* mutant. The *Hc* (G217B) *cbp1* mutant is unable to lyse macrophages after intracellular infection [32, 33]. A second phenotype of the *Hc cbp1* mutant is a delay in intracellular growth after macrophage infection, although ultimately the *cbp1* mutant reaches high levels of intracellular fungal burden equivalent to or exceeding that of wild-type *Hc* [32, 33]. We have previously shown that expression of the *Paracoccidioides americana* (Pb03) Cbp1 homolog in the *Hc* (G217B) *cbp1* mutant can restore macrophage lysis [32]. We expressed five of the newly detected *Emergomyces* Cbp1 homologs (*Es*. *africanus*, *Es*. *orientalis_1*, *E*. *crescens*, *Es*. *pasteuriana_1* and *Es*. *pasteuriana_2 Cbp1*) in the *Hc* G217B *cbp1* background to see if these homologs can complement either the ability of *Hc* to lyse macrophages or grow intracellularly without delay. To monitor for production and secretion of each of the *Emergomyces* homologs, we subjected concentrated culture supernatants from multiple isolates expressing each of the Cbp1 homologs to SDS-PAGE to monitor for the presence of a prominent Cbp1 band. We found that all five homologs were found in *Hc* culture supernatants in comparable amounts when expressed under the control of the *Emergomyces* native signal peptide (Fig 3A). Their identity was also confirmed with mass spectrometry of chymotrypsin digested peptides from SDS-PAGE gel bands (S1 Table).

To determine if expression of any of these homologs could complement the phenotypes of the *Hc* G217B *cbp1* mutant, we infected primary murine bone marrow-derived macrophages (BMDMs) with our cohort of heterologous expression strains and monitored for the release of lactate dehydrogenase into the supernatant as a measure of host cell lysis. The *Hc cbp1* mutant fails to lyse BMDMs and also exhibits an intracellular growth delay before it undergoes replication during infection. The Pb03 homolog was able to fully restore the lytic capability of the *cbp1* mutant (Fig 3B) but did not complement the growth delay as measured by intracellular CFU counts (Fig 3C). (In previous published work, we observed that the Pb03 homolog was able to complement the growth delay [32] and the reason for this discrepancy is unclear.) Surprisingly, none of the *Emergomyces* Cbp1 homologs were able to restore the ability of the *Hc* G217B *cbp1* mutant to lyse macrophages (Fig 3B). The *Emergomyces* homologs showed variable ability to support intracellular growth in macrophages: *cbp1* mutant cells expressing the *Es*. *orientalis* Cbp1 grew on par with the *Hc cbp1* mutant, cells expressing the *E*. *crescens* Cbp1 never grew well inside macrophages, and the *Es*. *africanus*, *Es*. *pasteurianus-1 and -2* alleles conferred variable growth (Fig 3C), suggesting some of these variants could interfere with intracellular growth.

To assess which molecular differences between Cbp1 homologs might correlate with differences in function, we created a series of chimeric Cbp1 protein constructs (S3A and S3B Fig). Of the *Emergomyces* Cbp1 homologs, we chose *E*. *crescens* Cbp1 as a template because it had the highest percent identity to *Hc* G217B Cbp1. Based on the alanine scan data described above, we identified key amino acids in *Hc* G217B Cbp1 that are required for macrophage lysis [32] (S3A Fig). A subset of the analogous amino acids in *E*. *crescens* Cbp1 were changed to the corresponding G217B *Hc or* Pb03 Cbp1 residues. These residues were selected based on the following criteria: whether their sidechains were facing the external surface, whether they were necessary for lysis based on the alanine scan data, and whether there was a significant change in either polarity, charge, or size as compared to *Hc* G217B Cbp1. Four chimeric constructs were ultimately generated that either exchanged the 1) entire N-terminal helix, 2) only the residues necessary for lysis in the N-terminal helix, 3) the residues necessary for lysis in the loops and helices of the C-terminal helical bundle, or 4) a combination of constructs 2 and 3 (S3A Fig). These chimeric Cbp1 proteins were expressed in the *Hc* G217B cbp1 mutant background. We found that the chimeric proteins were expressed and secreted (Fig 3D), suggesting that they were properly folded, but none could restore macrophage lytic capability (Fig 3E). Interestingly, while expression of *E*. *crescens* Cbp1 seemed to block intracellular growth (Fig 3C), chimeras of *E*. *crescens* Cbp1 with either the *Pb03* or G217B N-terminal residues necessary for lysis or *E*. *crescens* Cbp1 with the G217B C-terminal helical bundle displayed only a mild growth defect (Fig 3F).

### *Es*. *africanus* Cbp1 is unable to cause macrophage lysis despite replicating intracellularly

**Fig 4:**
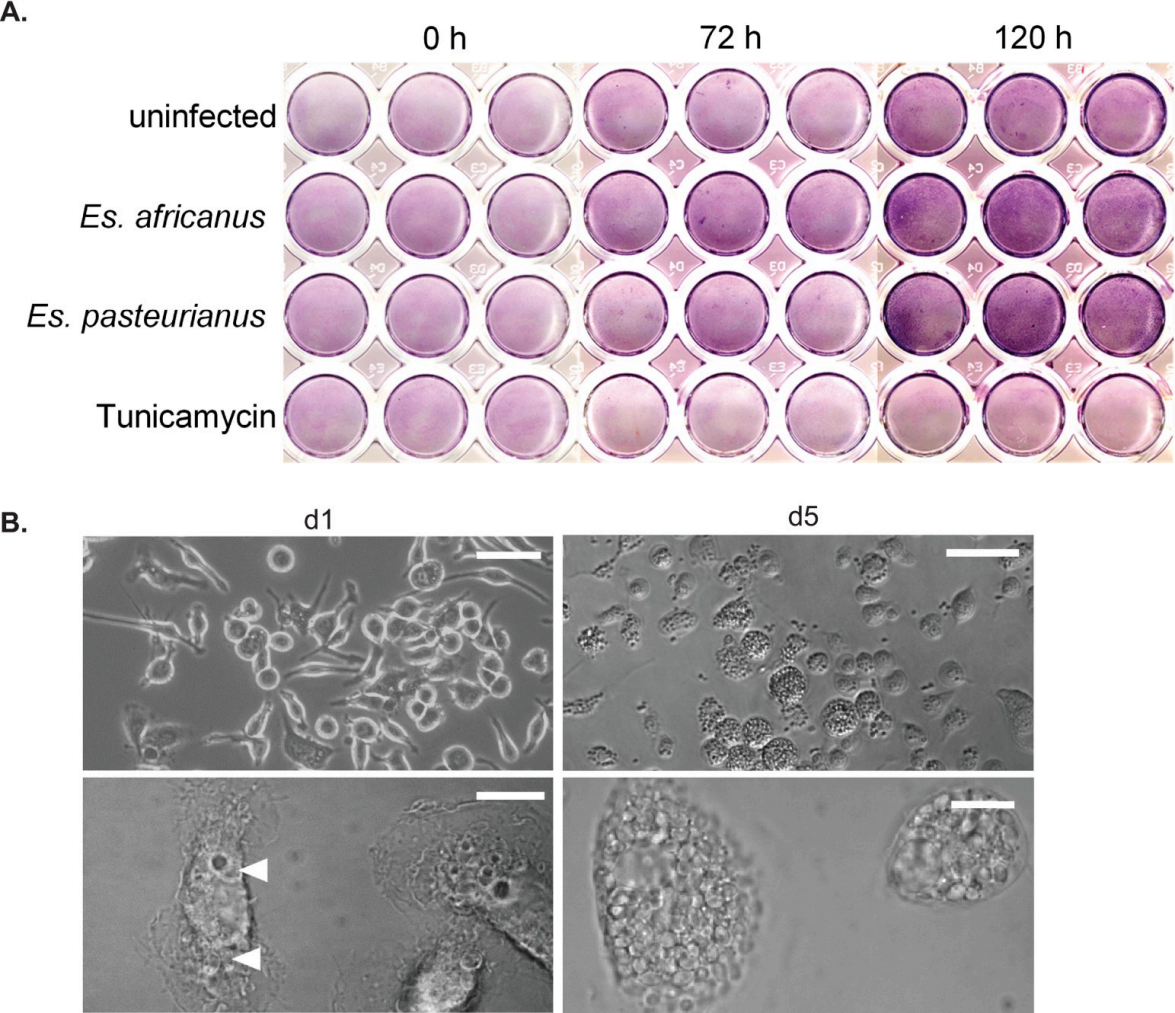
*Es*. *africanus* and *Es*. *pasteurianus* yeast can replicate intracellularly in macrophages but are unable to cause lysis. A. *Es*. *africanus* and *Es*. *pasteurianus* yeast were used to infect BMDMs at an MOI of 1 as described in the Materials and Methods and the macrophage monolayers were stained with Rapi-Diff Stain II Set to qualitatively measure monolayer clearance at 0, 72 and 120 hours post infection. B. DIC images of BMDMs infected with wildtype *Es*. *africanus* at an MOI of 1 at one day post-infection (left) or five days post infection (right). The scale bar represents 100 μm for the top images and 10 μm for the bottom (zoomed-in) images. Arrowheads indicate intracellular yeasts, which are marked on d1 but not on d5 when the macrophage is filled with yeast cells.

To determine if the lack of lysis caused by *Emergomyces* Cbp1 homologs is reflective of the *Emergomyces* natural infection of primary macrophages, we infected BMDMs with *Es*. *africanus* or *Es*. *pasteurianus* wildtype yeast at an MOI of 1. Macrophage lysis was monitored by examining the clearance of a macrophage monolayer. We observed no lysis of infected macrophages over the course of infection (Fig 4A). Tunicamycin treatment was used as a positive control for BMDM lysis (Fig 4A). Despite the lack of macrophage lysis, intracellular replication of *Es*. *africanus* was observed, and macrophages became filled with yeast cells over the course of the infection (Fig 4B). This behavior is strikingly similar to how the *Hc cbp1* mutant grows intracellularly to high levels but cannot lyse host cells [33]. The lack of macrophage lysis in *Hc* expressing the *Emergomyces* Cbp1 homologs is consistent with the inability of *Es*. *africanus* and *Es*. *pasteurianus* to lyse macrophages during infection. *Es*. *africanus* and other *Emergomyces* species are still capable of causing systemic disease in humans [36, 37] suggesting that these fungi may rely on virulence factors other than Cbp1.

### *Hc* G217B and Pb03 Cbp1 enter the cytosol during macrophage infection

**Fig 5:**
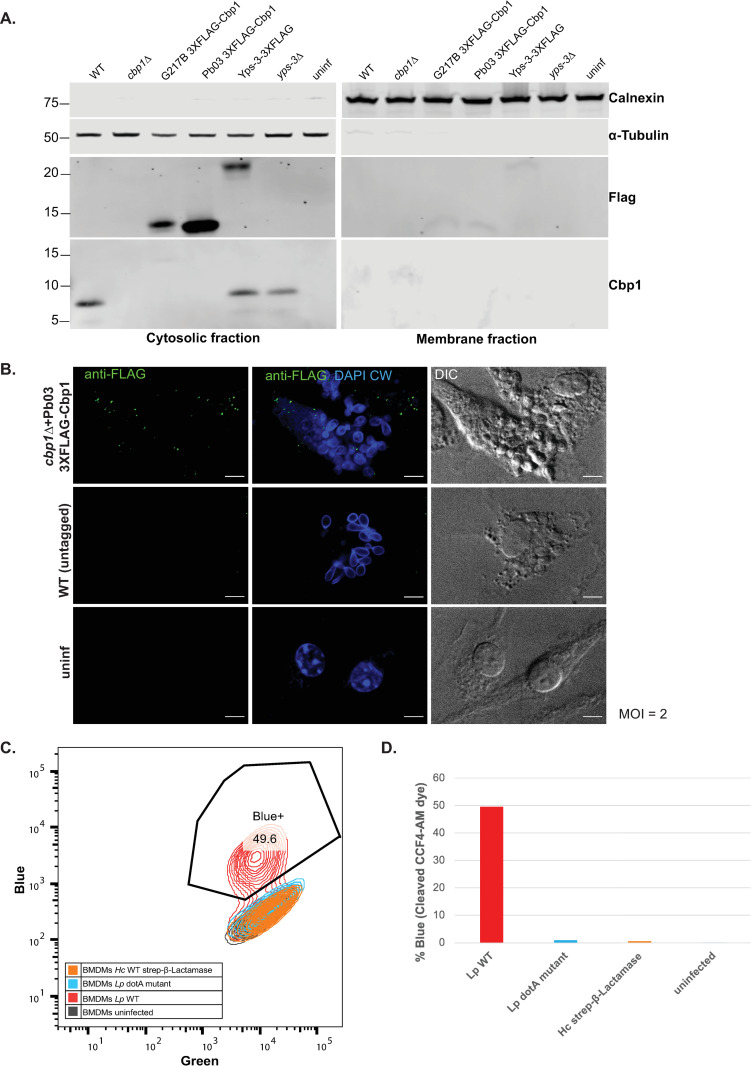
*Hc* and *Pb03* Cbp1 enter the macrophage cytosol during infection despite lack of general permeabilization of the *Hc-*containing phagosome. A. BMDMs were mock-infected (uninf) or infected with either the “WT” strain (*Hc* G217B *ura5*△ carrying a *URA5* vector control), the *cbp1* mutant (*Hc* G217B *ura5*△ *cbp1*△) carrying either the vector control or G217B Cbp1 with 3XFLAG (G217B Cbp1 3XFLAG), Pb03 Cbp1 with 3XFLAG (Pb03 Cbp1 3XFLAG), WT G217B *ura5*△ carrying Yps-3 3XFLAG (Yps-3 3XFLAG), or the *Hc* G217B *ura5*△ *yps3*△ mutant (*yps3*△). Macrophage lysates were subjected to fractionation to separate cytosolic and membrane fractions, followed by SDS-PAGE and Western blotting using anti-Calnexin (marking the endoplasmic reticulum), anti-α-Tubulin (marking the cytosol), anti-FLAG or anti-Cbp1 antibodies. B. BMDMs were either mock-infected or infected at an MOI of 2 with *Hc* G217B *ura5*△ *cbp1*△ expressing the WT untagged Cbp1(WT) or the *cbp1* mutant carrying the Pb03 Cbp1 tagged with 3XFLAG. At 48 hours post infection, cells were fixed, stained with DAPI and Calcofluor White, and subjected to indirect immunofluorescence with the FLAG antibody. Scale bar represents 10 μm. C. BMDMs were either mock-infected or infected with WT *Hc* (*Hc* G217B *ura5*△ carrying a *URA5* vector control) or *Hc expressing* β-Lactamase at an MOI of 2 for 24 hours or infected with a WT *Legionella pneumophila* (flaA△, BlaM-RalF) or the *dotA* mutant (dotA△, BlaM-RalF, flaA△) at an MOI of 100 for 4 hours. The BMDMs were then loaded with the CCF4-AM FRET probe and then monitored for probe cleavage as denoted by a shift from green to blue by flow cytometry. D. The percentage of the total cell population that showed blue fluorescence for each sample type in panel C is displayed.

To further probe the mechanism of Cbp1 function, we investigated its subcellular localization during *Hc* infection. Once *Hc* is phagocytosed by macrophages, it begins to replicate intracellularly within a modified phagosomal compartment that fails to fuse with degradative lysosomes and maintains a neutral pH [25, 48, 49]. We have previously shown that *Hc* infection triggers a cytosolic stress response inside of macrophages known as the Integrated Stress Response (ISR) that is dependent on Cbp1 [32]. As in the case of *Hc* infection, if the cytosolic stress remains unresolved, the macrophage undergoes apoptotic cell death. Since Cbp1 seems to be a crucial secreted virulence factor and is required to cause a host cell cytosolic response, we hypothesized that Cbp1 gains access to the macrophage cytosol during infection. A Cbp1-GFP fusion was previously shown to localize to the *Hc*-containing phagosome [28], but localization of the native Cbp1 during infection has not been determined and it is unknown whether the Cbp1-GFP fusion is functional. Almost all attempts at tagging *Hc* Cbp1 render it non-functional, and peptide antibodies generated against Cbp1 recognize only the denatured form of the protein, precluding localization by indirect immunofluorescence. To overcome these limitations, we fractionated infected macrophages and monitored Cbp1 accumulation in the macrophage cytosol fraction vs. the membrane fraction, which includes the contents of intracellular vesicles. We separated the cytosolic fraction from the membrane fraction (validated by probing for alpha-tubulin, and calnexin respectively) and Cbp1 localized exclusively to the cytosolic fraction, suggesting it exits the *Hc*-containing phagosome during infection (Fig 5A). We checked that the *Hc* yeast themselves are not rupturing during the fractionation protocol by confirming that the *Hc* transcription factor Ryp1 is present only in the fraction that contains whole *Hc* yeast, which are separated from the lysates along with the nuclear fraction prior to ultracentrifugation (S4A and S4B Fig). Additionally, to confirm that small endosomal compartments remained intact during the fractionation process, we determined that LAMP1 was not detectable in the cytosolic fraction (S4B Fig). Using a N-terminal FLAG-tagged Pb03 Cbp1 homolog that retains its lytic capability during infection (S5A Fig), we confirmed the cytosolic localization of Pb03 Cbp1 via this subcellular fractionation approach (Fig 5A). Interestingly, the analogous tagged version of *Hc* G217B Cbp1 is non-functional (S5B Fig) but nonetheless localizes to the macrophage cytosol during infection, suggesting cytosolic localization alone is not sufficient to cause lysis of the host macrophage (Figs 5A and S5). The functional *Pb03*-3XFLAG Cbp1 homolog appeared in a punctate pattern throughout the infected macrophage cytosol and occasionally overlapped with the *Hc*-containing phagosome, suggesting it could be accumulating and aggregating in the cytosol during infection (Fig 5B).

To assess whether Cbp1 could be accessing the cytosol because the *Hc*-containing phagosome is leaky, we monitored the integrity of the phagosome using a CCF4-AM assay which has been well established as a measurement of vacuolar rupture caused by bacterial pathogens such as *Shigella flexneri* and *Mycobacterium tuberculosis* [50]. CCF4-AM is a FRET probe consisting of two fluorophores attached by a β-lactam linkage. The intact probe emits green fluorescence but in the presence of β-lactamase, the probe is cleaved, resulting in blue fluorescence. *Legionella pneumophila (Lp)*, which triggers vacuolar permeabilization in a manner dependent on the Type IV secretion system [51], was used as a control. We loaded the CCF4-AM dye into the cytosol of macrophages that were either uninfected or infected with (1) WT *Lp* expressing β-lactamase, (2) a *dotA* mutant of *Lp* that lacks the Type IV secretion system and cannot secrete β-lactamase or permeabilize the vacuolar membrane, or (3) *Hc* secreting a functional β-lactamase (Figs 5C and 5D and S4C). We observed a robust blue shift of CCF4-AM fluorescence in macrophages infected with WT *Lp*, whereas no shift was observed in the other samples. These data indicate that the *Hc*-containing phagosome is not broadly permeabilized.

### *Hc* Cbp1 forms a complex with Yps-3, another known *Hc* virulence factor that is also in the macrophage cytosol

**Fig 6:**
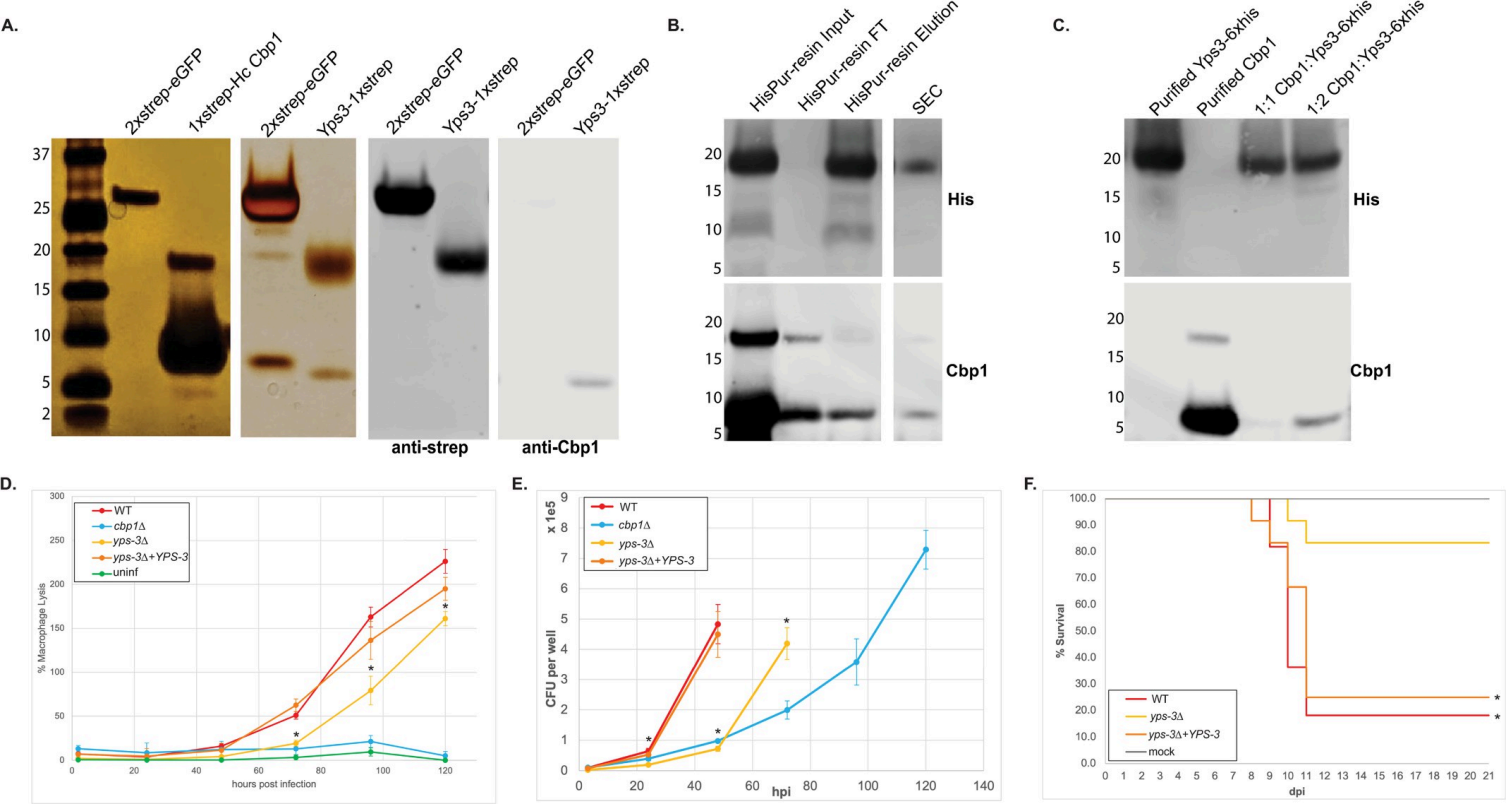
*Hc* G217B Cbp1 forms a complex with Yps-3. A. *Hc* culture supernatants from the *Hc* G217B *ura5*^*-*^ strain carrying a plasmid expressing 2xstrep enhanced GFP (2xstrep-eGFP) or Yps3-1xstrep, and the *Hc* G217B *cbp1* mutant expressing either G217B 1xstrep-Cbp1 or Pb03 2xstrep Cbp1 were subjected to StrepTactin affinity purification. Eluates were analyzed by SDS-PAGE followed by silver staining. The pulldowns of Yps-3-1xstrep and 2xstrep-eGFP were confirmed with SDS-PAGE followed with Western blot analysis with both anti-strep and anti-Cbp1 antibodies. B. *Hc* culture supernatant from the Yps-3-6XHis strain was subjected to SDS-PAGE followed by Western blotting with either anti-His or anti-Cbp1 antibodies. The left-hand panel shows input, flow-through (FT) and elution from the HisPur resin whereas the right-hand panel shows the fractions containing both Yps3 and Cbp1 from a size exclusion column (SEC). C. Purified Yps3-6XHis and purified G217B Cbp1 were either subjected separately to SDS-PAGE and Western blotting (lanes 1 and 2) or mixed first at defined molar ratios and then isolated by Ni-NTA pulldown of Yps-3-6XHis (lanes 3 and 4). D. BMDMs were either mock-infected (uninf) or infected at an MOI = 1 with G217B *ura5-* transformed with a *URA5* vector (WT), the *cbp1* mutant transformed with a *URA5* vector, G217B *yps-3Δ* transformed with a *URA5* vector, or the G217B *yps-3Δ* mutant transformed with wild-type *YPS-3*. LDH release was calculated at multiple timepoints post-infection. E. Intracellular growth of WT, cbp1*Δ*, *yps-3Δ*, yps-3*Δ+YPS-3* yeast was determined from BMDM infection as described in the Materials and Methods. CFU counts were terminated at 48 hpi for the WT *Hc* infection and 72 hpi for the *yps-3Δ* infection because the extent of host-cell lysis made it difficult to distinguish between intracellular and extracellular growth of *Hc*. F. Kaplan-Meier survival curves of C57BL/6 mice mock infected (uninf, n = 3) or infected with 1x10^6^ *Hc* yeast (n = 11–12). Asterisk denotes p-value <0.05 by log rank test.

To see if Cbp1 acts alone or as a part of complex of fungal proteins, we isolated 1xstrep-tagged G217B Cbp1 (S5C Fig) from *Hc* culture supernatants and determined which *Hc* proteins associate with Cbp1 by mass spectrometry (Fig 6A). The 1xstrep-tagged G217B Cbp1 allele retains partial lytic function during macrophage infection (S5C Fig). In the eluates of the 1xstrep *Hc* G217B Cbp1 pulldown we observed a prominent band around 17–20 kDa that was absent in the control pull down of 2xstrep-eGFP. The identity of this band was later confirmed by mass spectrometry as *Hc* virulence factor Yps-3 [34]. Conversely, Cbp1 co-purified with a pulldown of Yps-3-1xstrep from *Hc* culture supernatants (Fig 6A). Additionally, our fractionation experiments demonstrated that, like Cbp1, Yps-3 is able to enter the macrophage cytosol during *Hc* infection (Fig 5A).

To determine if Yps-3 and Cbp1 form a complex, we purified C-terminally tagged Yps3-6xhis from culture supernatants using a cobalt resin and observed that Cbp1 co-purifies with Yps-3 (Fig 6B). To isolate pure Yps3-6xhis, we used a cation exchange column, but a large portion of Yps-3 was present in the flow through of the cation exchange column along with Cbp1. To confirm that the proteins are truly in a complex, we passed the flow through over a sizing column and discovered that Yps-3 and Cbp1 were present in the same fractions, suggesting they remained in a tight complex (Fig 6B). Additionally, to examine the ability of purified proteins to directly interact, we mixed purified Yps3-6xhis and G217B Cbp1 in molar ratios of 1:1, 1:2, 2:1, 5:1 and isolated the 6xHis tagged Yps3 with a Ni-NTA resin. We found that the molar ratio of 1:2 Cbp1:Yps-3 yielded the most robust interaction between the two proteins (Figs 6C and S6).

Previously Yps-3 RNAi knockdown had shown no yeast phenotype in a macrophage cell line [34]. Since RNAi may not be definitive, to further characterize the role of Yps-3 in macrophage infection we generated a CRISPR deletion mutant of *YPS-3* (S7A and S7B Fig) [52, 53]. We found that the *yps-3Δ* mutant has a partial lysis defect in BMDMs (Fig 6D) despite the presence of Cbp1 in the cytosol of macrophages infected with this mutant (Fig 5A). We next examined the ability of the *yps-3Δ* mutant to grow intracellularly within macrophages (Fig 6E). The mutant displayed a delay in intracellular growth, although the defect was not as severe as that of the *cbp1* mutant. Additionally, unlike the *cbp1* mutant [33], the *yps-3Δ* mutant had a mild growth defect in culture (S7C Fig). We previously established that Cbp1 is required for *Hc* to trigger the ISR during macrophage infection [32]. To determine if *YPS-3* is also required for *Hc* to trigger the ISR, we examined transcriptional induction of Tribbles 3, a reporter of the ISR response (S7D Fig). We observed robust ISR induction during infection of BMDMs with wild-type or *yps-3Δ* mutant *Hc*, but not with the *cbp1* mutant, indicating that Yps-3 is not required for the ability of Cbp1 to trigger the ISR. Taken together, these data indicate that Yps-3 is required for robust lysis of infected macrophages, perhaps because it is required for optimal intracellular accumulation of yeast cells and/or it potentiates an activity of Cbp1 that is independent of ISR induction.

RNA interference strains targeting Yps-3 were previously used to implicate Yps-3 in organ colonization in the mouse model of infection [34].To further assess the role of Yps-3 in the mouse model of histoplasmosis, we infected mice with wild-type *H*. *capsulatum* (G217B *ura5Δ* + *URA5*), the *yps-3* mutant (*yps-3Δ* + *URA5*) or the complemented stain (*yps-3Δ* + *YPS-3*) as described in Materials and Methods. We observed a virulence defect in the *yps-3* mutant compared to the wild-type and complemented strains, indicating that Yps-3 plays an important role in the ability of *Hc* to promote disease.

## Discussion

**Fig 7:**
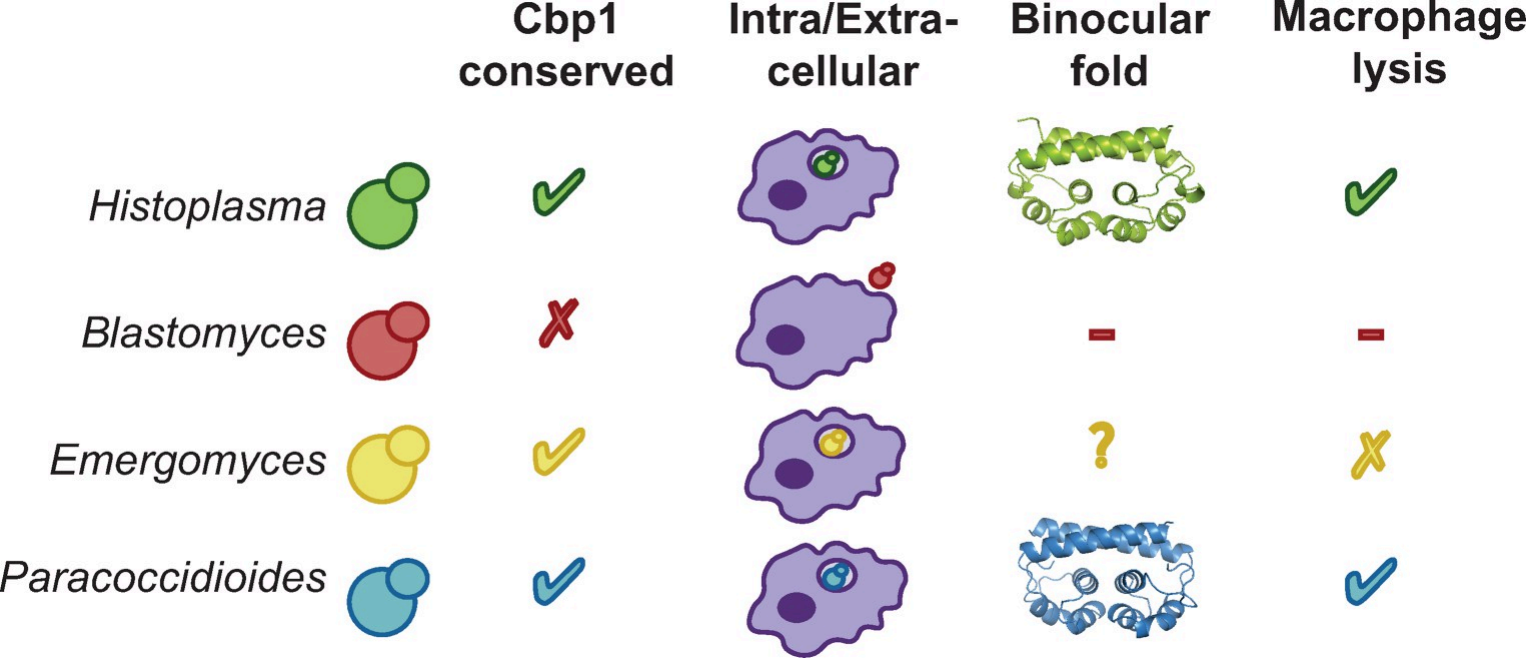
Model figure showing the conservation of Cbp1 sequence, structure, and lytic function in Ajellomycetacea species that are human fungal pathogens. Based on predicted protein homology and syntenic regions of the gene, homologs of Cbp1 were identified in the genomes *Histoplasma*, *Emergomyces*, *Emmonsia*, and *Paracoccidioides* species but not *Blastomyces*, correlating with its largely extracellular lifestyle relative to the other organisms. A novel structural fold was conserved between the *Histoplasma* and *Paracoccidioides* Cbp1 homologs despite divergence of their primary amino acid sequence. The *Paracoccidioides* Cbp1 homolog is capable of causing lysis of infected macrophages when expressed heterologously in G217B *Hc*. In constrast, none of the newly identified *Emergomyce*s or *Emmonsia* homologs could trigger macrophage lysis when expressed heterologously in G217B *Hc*. These results are consistent with our observation that *Emergomyces* species are unable to lyse out of BMDMs.

Macrophages are innate immune cells that are critical for the early detection and killing of infectious microbes. *Hc* is an intracellular pathogen that evades macrophage anti-microbial mechanisms, thereby surviving and replicating within a modified phagosomal compartment. Here we focus on a secreted *Hc* effector, Cbp1, that is critical for manipulating the macrophage response. Cbp1 is absolutely required for macrophage lysis during *Hc* infection. We identified all existing Cbp1 homologs in the fungal kingdom and interrogated these proteins for their ability to trigger macrophage lysis when expressed in a *Hc cbp1* mutant. These experiments revealed that only *Hc* and *Pb* carry “lytic” Cbp1 alleles, whereas closely related *Emergomyces* Cbp1 variants cannot promote macrophage lysis during *Hc* infection. Similarly, *Emergomyces* species were unable to lyse macrophages during native infections, suggesting that the *Emergomyces* Cbp1 proteins are non-lytic. Interestingly, although we could not solve the structure of *Emergomyces* Cbp1 proteins, the crystal structure of *Hc* and *Pb* Cbp1 proteins revealed a novel “binocular” fold. These data are summarized in Fig 7, which highlights that among these evolutionarily related fungi, the intracellular pathogens have maintained Cbp1, which promotes lysis for a subset of these organisms.

Our previous work revealed that *Hc* Cbp1 is absolutely required for triggering an Integrated Stress Response (ISR) in the macrophage cytosol during *Hc* infection. When we mutagenized Cbp1 to yield alleles that were partially lytic or completely defective for lysis, we observed little or no ISR induction, respectively, indicating a correlation between the ability of Cbp1 to trigger the ISR and lyse macrophages [32]. Here we show for the first time that *Hc* Cbp1 accesses the macrophage cytosol during infection, suggesting that the site of action of Cbp1 is in the cytosol of the host cell, where it presumably causes a stress that activates the ISR. Additionally, we show that Cbp1 binds another known *Hc* virulence factor, Yps-3. Previous work in the literature showed that Yps-3 localizes to the *Hc* cell wall and is released into culture supernatant. RNA interference strains targeting Yps-3 were used to show a role for Yps-3 in organ colonization in the mouse model of infection but no defect in macrophage lysis was observed at the single timepoint examined [34]. Interestingly, we show here that deletion of Yps-3 results in diminished host-cell lysis, suggesting that Yps-3 could help potentiate the function of Cbp1.

How Cbp1 and Yps-3 transit from the phagosome to the macrophage cytosol is not yet clear. Perhaps the *Hc-*containing phagosome is leaky, thereby allowing small fungal proteins (but not proteins of the size of β-lactamase) to access the cytosol. Alternatively, *Hc* is known to secrete copious amounts of extracellular vesicles that contain varied cargo of *Hc* proteins [54, 55]. These virulence factors could enter the cytosol because exosomes, or even multi-vesicular bodies, fuse with the phagosome membrane and release their contents directly into the cytosol. Once in the reducing environment of the host cytosol, perhaps Cbp1 changes its conformation and triggers the ISR. We observed that a tagged allele of the Pb03 Cbp1 (Pb03*-*3XFLAG Cbp1) was distributed throughout the cytosol in a punctate pattern. We speculate that Cbp1 could be aggregating in the cytosol and triggering cellular stress, and future work will focus on the mechanism of action of the Cbp1-Yps-3 complex in triggering host-cell death.

We observed that Cbp1 has a high rate of non-synonymous relative to synonymous sequence changes, consistent with positive selection. Since Cbp1 is present in all of the genomes of the Ajellomycetaceae human fungal pathogens except *Blastomyces* species, it is likely that Cbp1 arose at the base of the Ajellomycetaceae tree and was subsequently lost only in *Blastomyces*. Notably, *Blastomyces* yeast cells are largely extracellular during infection of mammals, in contrast to *Histoplasma* and other Ajellomycetaceae, suggesting that Cbp1 could be an adaptation to an intracellular lifecycle. Interestingly, despite its lack of Cbp1, *Blastomyces* has retained a Yps-3 homolog, *Blastomyces* Adhesin 1 (Bad1). Bad1 mediates binding of *Blastomyces* to host cells and plays an immunoregulatory role during infection [39, 56–59]. In the case of *Hc*, not all strains produce Yps-3 protein [34]. In the *Hc* G217B background, our data suggest that Yps-3 acts with Cbp1 to promote macrophage lysis whereas in other *Hc* strains, it may be the case that Cbp1 acts in a Yps-3-independent manner. Additionally, future experiments are needed to elucidate Cbp1-independent roles of Yps-3 in the virulence of G217B.

*Hc* Cbp1 is critical for macrophage lysis and dissemination [32, 33]. Pb18 Cbp1 is highly expressed in *Paracoccidioides* Pb18 yeast cells [60] and Pb03 Cbp1 is capable of triggering host-cell lysis when expressed in the *Hc* G217B *cbp1* mutant background, but whether any *Paracoccidioides* species utilize Cbp1 during infection is yet to be determined. In contrast, *Emergomyces* species have Cbp1 in their genomes but heterologous expression of these Cbp1 alleles in *Hc* suggests that they are non-lytic. This observation correlates with the natural infection of primary BMDMs by *Es*. *africanus* and *Es*. *pasteurianus*, characterized here for the first time. We observed that *Es*. *africanus* and *Es*. *pastuerianus* undergo intracellular replication within macrophages but cannot lyse them, much like the *Hc* G217B *cbp1* mutant. It is unclear how the absence of a lytic Cbp1 affects *Emergomyces* pathogenesis, and future work elucidating the molecular virulence strategies of these pathogens will be key. We found that expression of certain heterologous Cbp1 variants resulted in decreased accumulation of intracellular *Hc* yeast cells during macrophage infection. The reason for lower intracellular CFUs is unknown, but it may be that expression of these heterologous variants prevents nutrient acquisition by intracellular yeasts.

The analysis of *Hc* H88 and Pb03 Cbp1 protein structures revealed a novel fold that was highly distinct from the previously published NMR G186AR Cbp1 structure. Our protein purification strategy was non-denaturing, which contrasts to the methodology used for the NMR structure. It is possible that Cbp1 can fold differently depending on its environment; for example, perhaps the reducing environment of the macrophage cytosol triggers a conformational change in Cbp1 protein. When we compared the two new structures of Pb03 and H88 Cbp1, we found that charge was not distributed similarly across the proteins. The bottom of the C-terminal helices that form the core four-helix bundle of the Pb03 Cbp1 are much more negative relative to the *Hc* H88 Cbp1 homolog. Interestingly, only the Pb03 (and not H88) structure shows partially negative N-terminal helices. In addition, when we overlaid the previously published alanine scan of G217B Cbp1 over the two new structures, we found that the majority of the residues necessary for optimal lysis are distributed in the N-terminal helix, suggesting that this region of the protein mediates host-cell death. However, when we swapped the 1^st^ helix of the non-lytic *E*. *crescens* Cbp1 allele with the corresponding helix from either G217B or Pb03 Cbp1, the resulting chimeric protein was not lytic, suggesting that other regions of the protein may affect folding and/or function. Nonetheless, the concordance between the alanine scan phenotypes and the location of the residues in the crystal structure gives strong support to our analysis of the Cbp1 fold and indicates that our structural work on Cbp1 is a critical step in understanding how intracellular fungal pathogens manipulate host cell viability.

## Materials and methods

### Ethics statement

All animal work was approved under UCSF Institutional Animal Care and Use Committee protocol # AN181753-02B.

### Primers

Primers used in this study are described in Table 2.

**Table 2:** Primers used in this study.

Primer name	Sequence	Primer use
DAZ296	AGCACCCAGTCGTTCGTTC	Primer external to Yps-3 CDS to monitor for CRISPR cleavage. Forward primer to be used with DAZ297.
DAZ297	GCACACAAGATGTTTCCCTATGTCCG	Primer external to Yps-3 CDS to monitor for CRISPR cleavage. Reverse primer to be used with DAZ296.
DAZ317	ACAACTACGACATCTACAAGCAATTCCGT	Internal primer in Yps-3 CDS to monitor for CRISPR cleavage. Forward primer to be used with DAZ297.
DAZ345	AAATCGATCTCAACCCTCCTCCTCCTT	Internal primer in Yps-3 CDS to monitor for CRISPR cleavage. Forward primer to be used with DAZ346.
DAZ346	CAGTGTTTATAAAGCGGAACCTCTTGGCA	Internal primer in Yps-3 CDS to monitor for CRISPR cleavage. Forward primer to be used with DAZ345

### Determining the conservation of Cbp1 in Ajellomycetaceae genomes and generating protein alignments

The Cbp1 protein alignment from Fig S3 of [32] was extended as follows: First, we used HMMer 3.1b2 [61] to build an HMM from the previous alignment and used it to search for homologs in the following annotated protein sets, downloaded from Genbank: *Emergomyces africanus* (GCA_001660665.1), *Emergomyces pasteuriana* (GCA_001883825.1), *Emmonsia parva* (GCA_001014755.1), *Blastomyces percursus* (GCA_001883805.1), *Paracoccidioides venezuelensis* Pb300 (GCA_001713645.1) and Paracoccidioides restrepiensis *PbCNH* (GCA_001713695.1). This yielded single homologs in *Es*. *africanus*, *Pb300*, and *PbCNH*, two homologs in *Es*. *pasteuriana*, and no homologs in *E*. *parva* or *B*. *percursus*, which is consistent with loss of *CBP1* in genus Blastomyces. The exon annotations of the five new homologs were refined based on the previous protein alignment. Four of the new homologs are syntenic to the previously known Cbp1s and share the conserved two intron structure. The remaining homolog, from *Es*. *pasteuriana*, is in a distinct genomic location and has three introns. TBLASTN from NCBI BLAST 2.6.0 [62] was then used to search the full set of Cbp1 protein sequences against the unannotated *Emergomyces orientalis* genome (GCA_002110485.1), yielding one hit orthologous to the conserved two intron Cbp1 sequence and one hit orthologous to the three intron *Es*. *pasteuriana* paralog. Exons were annotated for both *Es*. *orientalis* homologs based on the existing protein alignment, as above. All sequences were then aligned with PROBCONS 1.12 [63] to yield the final alignment. The *Es*. *africanus* Cbp1 had a small 7-amino acid insertion that was not present in the other orthologs.

### Species tree generation

Full length midasin sequences were inferred by using NCBI TBLASTN 2.6.0 [62] to search the G217B predicted sequence HISTO_DA.Contig93.Fgenesh_Aspergillus. 103.final_new against each genome of interest, retaining the top non-redundant hits spanning the full protein. An HMM was built from the initial TBLASTN alignments with hmmbuild (HMMer 3.1b2) [61], all hits were realigned to the HMM with hmmalign (HMMer 3.1b2), and the resulting multiple alignment was refined with PROBCONS 1.12 [63]. A phylogenetic tree was inferred from the ungapped positions of the PROBCONS multiple alignment with IQTREE 1.5.3 [64].

### PAML analysis of positive selection of Cbp1

The full length CBP1 protein alignment was cut to the 11 CBP1 orthologs (dropping the paralogs from *Es*. *pasteuriana* and *Es*. *orientalis*) and was converted to a nucleotide alignment by replacing amino acids with their cognate codons from the respective genome sequences. The corresponding evolutionary tree was taken from the midasin-based phylogeny, cutting the tree to just species with CBP1 and dropping branch lengths. CODEML from PAML 4.9 [41] was used to fit the nucleotide alignment and tree topology to four evolutionary models (M1a: neutral, M2a: positive selection, M7: beta, and M8: beta and omega). Models with and without positive selection were then compared by using CHI2 from PAML 4.9 to perform chi squared tests on the corresponding log likelihood ratios from CODEML with two degrees of freedom. The corresponding p-values are reported in the text. Specific positions under positive selection with a posterior probability greater than 0.95 based on model M2a were identified using the Naive Empirical Bayes output from CODEML.

### BMDM culture conditions

Bone marrow derived macrophages (BMDMs) were isolated from the tibias and femurs of 6–8 week old C57BL/6J (Jackson Laboratories stock no. 000664) mice. Mice were euthanized via CO2 narcosis and cervical dislocation as approved under UCSF Institutional Animal Care and Use Committee protocol # AN181753-02B. Cells were differentiated in BMM (bone marrow derived macrophage media) which consists of Dulbecco’s Modified Eagle Medium, D-MEM High Glucose (UCSF Cell Culture Facility), 20% Fetal Bovine Serum (Atlanta Cat #: S11150, Lot #: D18043), 10% v/v CMG supernatant (the source of CSF-1), 2 mM glutamine (UCSF Cell Culture Facility), 110 μg/mL sodium pyruvate (UCSF Cell Culture Facility), penicillin and streptomycin (UCSF Cell Culture Facility) with 20% Fetal Bovine Serum (Atlanta Cat #: S11150, Lot #: D18043). Undifferentiated monocytes were plated in BMM that contains BMM for 7 days at 37°C and 5% CO_2_. Adherent cells were then scraped and frozen down in 40% FBS and 10% DMSO until further use. *Hc* cultures were grown in liquid *Histoplasma* macrophage media (HMM) using an orbital shaker or on HMM agarose plates [33].

### Generation of Hc strains

*H*. *capsulatum* strain G217B *ura5Δ* (WU15) was a kind gift from the lab of William Goldman (University of North Carolina, Chapel Hill). For all studies involving the *cbp1* and *yps-3* mutants, “wildtype” refers to G217B *ura5Δ* transformed with a *URA5*-containing episomal vector (pLH211), *cbp1* refers to G127Bura5 *Δcbp1*::*T-DNA* as previously described [33] transformed with the same *URA5*-containing episomal vector, and “complemented” strain refers to *G217Bura5Δcbp1*::*T-DNA* transformed with the *URA5*-containing plasmid bearing the wild-type CBP1 gene (pDTI22) as previously described [33]. The *Emergomyces/Emmonsia CBP1* coding sequences including their signal peptides (*Es*. *africanus*, *Es*. *orientalis*, *E*. *crescens*, *Es*. *pasteuriana_1*, *Es*. *pasteuriana_2)* and *E*. *crescens> G217B* or *E*.*crescens>Pb03* chimeric constructs (N-terminal helix residues, C-terminal loop residues, both C-terminal and N-terminal residues, 1^st^ helix swap) constructs were synthesized as gBlocks by Integrated DNA Technologies and cloned into pDTI22, replacing the G217B *CBP1* coding sequence but maintaining the flanking sequences. In contrast to the *Emergomyces/Emmonsia* expression constructs, the *P*. *americana* strain *Pb03* Cbp1 gene construct includes the *Hc* G217B Cbp1 signal peptide (instead of the *Pb03* signal peptide) fused to the mature Pb03 protein coding sequence flanked by the same regulatory sequences of pDTI22 as previously described [32]. All tagged Cbp1 expressing strains including 1xstrep-H*c G217B* Cbp1, 2xstrep-*Pb03* Cbp1, 3XFLAG-*Hc G217B* Cbp1, 3XFLAG-*Hc Pb03* Cbp1, were N-terminally tagged with the tag situated between the G217B Cbp1 signal peptide and the mature protein sequence and were flanked by the same regulatory sequences in pDTI22 and introduced into the Hc G217B cbp1 mutant strain. The *yps3* mutant was generated from the G217B *ura5Δ* parental strain transformed with the episomal plasmid pDAZ021 which contains a bidirectional H2Ab promoter driving Cas9 and two sgRNA cassettes targeting the sequences on both sides of the G217B Yps-3 gene [52]. Single colony isolates for PCR screened in the yps-3 genomic locus to look for excisions in the colony population and the isolates with the most likely edited band were struck out for further single colony isolation until an isolate with a clean excision between the two sgRNA target sites was detected. pBJ219 was subsequently lost from the mutant by growing the *Hc* yeast in the presence of exogenous uracil and then screening for plasmid loss. Subsequently, the G217B *ura5Δ yps3Δ* was transformed with the *URA5-*containing episomal plasmid (pLH211) and a URA5-containing complementation vector. The overexpression strains used to detect and purify Yps-3 were generated by transforming G217B *ura5Δ* parental strain with an episomal plasmid with each of the C-terminally tagged Yps-3 alleles (Yps-3-6xHistidine, Yps-3-2xstrep, Yps-3-3XFLAG) flanked by the Cbp1 promoter and CatB terminator. The control for the StrepTactin pulldowns was an N-terminally Twinstrep (iba Lifesciences) tagged enhanced GFP (eGFP) that was fused to the G217B Cbp1 signal peptide and cloned into the same episomal plasmid (pDTI22) as all the other Cbp1 constructs. For all Cbp1, Yps3, eGFP, and CRISPR construct plasmids, approximately 50 ng of PacI-linearized DNA was electroporated into the appropriate parental strain (G217B *ura5Δ*, G217B ura5*Δ cbp1*::*T-DNA*, or G217B ura5*Δ yps3Δ*) as previously described [33]. The plasmid pLH211 was used as a vector control. Transformants were selected on HMM agarose plates.

### Hc secreted protein detection in culture supernatants

To detect *Hc* secreted proteins (tagged Cbp1 alleles and Cbp1 homologs, tagged Yps-3 homologs, tagged 2xstrep-eGFP) in culture supernatants, 4–5 day *Hc* cultures were grown in liquid HMM and yeast were pelleted by centrifugation. The supernatants were subjected to filtration using 0.22 μm filters, and the resultant filtrates were concentrated using Amicon Ultra Centrifugal Filter Units with a 3 kDa cutoff (EMD Millipore). Protein concentration was quantified using the Bio-Rad protein assay (Bio-Rad Laboratories). Equal amounts of protein were separated by SDS-PAGE, and proteins were visualized by staining the gel with InstantBlue Coomassie Protein Stain (ISB1L –abcam 119211)

### Macrophage infections

Macrophage infections with G217B *Hc* strains were performed as described previously [33]. Briefly, the day before infection, macrophages were seeded in tissue culture-treated dishes. On the day of infection, yeast cells from logarithmic-phase *Hc* cultures (OD_600_ = 5–7) were collected, resuspended in BMM, sonicated for 3 seconds on setting 2 using a Fisher Scientific Sonic Dismembrator Model 100, and counted using a hemacytometer at 40X magnification. Depending on the multiplicity of infection (MOI), the appropriate number of yeast cells was then added to the macrophages. After a 2-hour phagocytosis period, the macrophages were washed once with d-PBS (PBS that is free of Ca^2+^ and Mg^2+^) to remove extracellular yeast and then fresh media was added. For infections lasting longer than 2 days, fresh media was added to the cells at approximately 48 hours post infection.

### Fractionation of Hc-infected macrophage lysates

2.5x10^7^ BMDMs were plated on a TC-treated 15-cm plates and allowed to adhere for at least 24 hrs. The BMDMs were then infected with *Hc* at an MOI = 5 and the infection was allowed to proceed for 24 hours. At 24 hours post infection, cells were collected by scraping without washing and spun down at 2500 rpm for 5 min to pellet the intact cells. The cell pellet was then resuspended in 500 μL of d-PBS (Ca^2+^ Mg^2+^ free), transferred to a 1.5 mL tube, and spun at 1000 rpm for 2 min. The cell pellet was then resuspended in 300 μL of homogenization buffer (150 mM KCl, 20 mM HEPES pH 7.4, 2 mM EDTA, cOmplete Mini Protease Inhibitor Cocktail tablet-Roche 04693124001). For the unfractionated sample, the cell pellet was resuspended in homogenization buffer with 1% TritonX-100. For the fractionated samples, the cell lysate was then gently passaged through a 27-gauge needle to disrupt only the plasma membrane and not any internal membranous compartments. The lysate was then spun at 3000 rpm for 5 minutes to pellet *Hc* cells and macrophage nuclei. The remaining lysate was then further clarified by centrifuging twice at 3000 rpm with removal of 275μL and 250 μL respectively to avoid contamination of the lysate with *Hc*. After the final low speed spin, 225 μL was placed in ultracentrifuge tubes (Beckman Coulter 349622), weighed, and loaded into a Beckman Coulter Fixed Angle Rotor TLA100.3. The samples were then spun at 45,000 rpm for 2 hours in a TL-100 tabletop ultracentrifuge to pellet the membranes. After the spin, 175 μL of supernatant, representing the cytosolic fraction, was transferred into a separate 1.5 mL tube. The remaining supernatant was discarded to prevent cross-contamination between the cytosolic and membrane fractions. The pellet, representing the membrane fraction, was resuspended in 225 μL of homogenization buffer with 1% TritonX-100. All samples were flash frozen in liquid nitrogen and stored at -80°C.

### Lactate dehydrogenase release assay

To quantify macrophage lysis, BMDMs were seeded (7.5 x 10^4^ cells per well of a 48-well plate) and infected as described above. At the indicated time points, the amount of LDH in the supernatant was measured as described previously [33]. BMDM lysis was calculated as the percentage of total LDH from supernatant of the uninfected wells with uninfected macrophages lysed in 1% Triton X-100 at the time of infection. Due to continued replication of BMDMs over the course of the experiment, the total LDH at later time points is greater than the total LDH from the initial time point, resulting in an apparent lysis that is greater than 100%.

### Intracellular replication

BMDMs were seeded (7.5x10^4^ cells per well of a 48-well plate) and infected in triplicate as described above. At the indicated timepoints, culture supernatants were removed and 500 μl of ddH2O was added. After incubating at 37°C for 15 min, the macrophages were mechanically lysed by vigorous pipetting. The lysate was collected, sonicated to disperse any clumps, counted, and plated on HMM agarose in appropriate dilutions. After incubation at 37°C with 5% CO_2_ for 12–14 days, CFUs were enumerated. To prevent any extracellular replication from confounding the results, intracellular replication was not monitored after the onset of macrophage lysis.

### Purification, crystallization and structural determination of Cbp1

2–3 L of G217B, G186AR, or H88 *Hc* or 2-3L of G217B *Hc* expressing *CBP1* from *Es*. *africanus*, *E*. *crescens*, or *P*. *americana* were grown at 37°C with 5% CO_2_ for 5 days. Culture medium was concentrated using an Amicon cell outfitted with a 5kDa molecular weight cutoff membrane. Concentrated medium was diluted 1:50 in MonoA buffer (20 mM Tris-HCl pH 7.5, 20 mM NaCl) and run through a HiTrapQ ion exchange column. Protein generally eluted at a 24% MonoB buffer concentration (20 mM Tris-HCl pH 7.5, 1 M NaCl). Eluted fractions were pooled and concentrated using a 3 kDa molecular cutoff spin concentrator. The final product was separated from remaining contaminants using a size exclusion approach with a Superdex 75 10/300 column run with SEC buffer (50 mM Tris-HCl pH 7.5, 150 mM NaCl). Purity of the final elution was verified by SDS-PAGE and concentrated using a 3 kDa MWCO spin concentrator. Pb03 Cbp1 was concentrated to 6 mg/mL and H88 Cbp1 was concentrated to 14 mg/mL. Crystallization trays were set up in sitting drop vapor diffusion trays at room temperature using 2 μL + 2 μL drops of protein and crystallization solution. Crystals of Pb03 Cbp1 formed in 0.05 M HEPES pH 6.5, 35% PEG 6000, and were seen after 24 hours and allowed to grow for 48 hours to reach full size. H88 Cbp1 crystallized in 0.02 M CoCl2, 0.2 M MES pH 6.5, 2 M Ammonium sulfate using the same setup as for Pb03 Cbp1, crystals were observed after 48 hours and reached full size in 1 week. To provide phase information, full sized Pb03 Cbp1 crystals were soaked for 2 hours in 10 mM potassium tetrachloroplatinate (II) from the Hampton Heavy Atom kit screen. Derivatized and native Pb03 Cbp1 crystals were cryoprotected using ethylene glycol. H88 Cbp1 crystals were cryoprotected using glycerol. All diffraction data were collected at ALS BL 8.3.1 on a Pilatus3 S 6M detector. Data processing and refinement was conducted in the CCP4 and Phenix programming suites. Datasets were processed using XDS and sailed using AIMLESS in the CCP4 suite. The structures were solved using Phaser in the Phenix crystallography suite. We obtained crystals for G217B and G186AR Cbp1s but were unable to resolve their diffraction patterns. For *Es*. *africanus* and *E*. *crescens* Cbp1 proteins, we were unable to generate useable crystals.

### BMDM infection with Emergomyces africanus and Emergomyces pasteurianus yeast

*Emergomyces africanus* (clinical isolate CBS 136260 [65, 66]) and *Emergomyces pasteurianus* (clinical isolate CBS140361 provided by Prof Nelesh Govender NICD, South Africa) were grown in 100% FBS for 5 days, followed by subculture for 3 days prior to infection to ensure yeast-phase growth. BMDMs were seeded in 8-well μ-Slide imaging slides (Cat. No. 80826, ibidi, Germany) at a density of 7.5 x 10^4^ cells/well in 0.375 ml BMM (DMEM, high glucose, GlutaMAX supplement, pyruvate (Thermo Fisher, Cat no. 10569010), 20% FBS (Thermo Fisher, Cat. no. 10270106), Penicillin-streptomycin (50 units/mL) (Thermo Fisher, Cat. no. 15140148), 20 ng/ml mCSF (R&D Systems, Cat no. 416-ML), and incubated at 37°C, 5% CO_2_ for 24 h. Yeast cells used for infection were washed twice by centrifugation and resuspension in PBS, counted using a hemocytometer, then the appropriate volume of yeast suspension was added to BMM. On day 0, BMDMs were infected by replacing the media with 0.375 ml yeast suspension in BMM, with a total of 7.5 x 10^4^ yeasts/well (MOI = 1). The BMDMs were fed on day 2 by removing 200 μL spent media from wells and replacing it with 200 μL fresh BMM. At the indicated time points, the macrophage monolayers were stained to evaluate the degree of cell lysis. This was done using the Rapi-Diff Stain Set (Clinical Sciences Diagnostics, South Africa) as follows: Media was removed from wells, and wells were washed once with 1 mL of PBS. 200 μL of methanol was applied for 5 minutes, then exchanged with 200 μL Solution I for 5 minutes (Eosin Y solution), then exchanged with 200 μL Solution II (Methylene blue and Azure A solution) for 5 minutes. The wells were then washed 3–4 times with 1 mL distilled water until the washes were colourless, and the plates were photographed. Tunicamycin (10 μg/ml) was used as a positive control for macrophage lysis.

### LDH assay sample collection of BMDMs infected with Es. africanus

BMDMs were seeded in 48-well tissue culture plates at a density of 1 x 10^5^ cells/well in 0.5 ml BMM, and incubated at 37°C, 5% CO_2_ for 24 h. Yeast cells used for infection were washed twice by centrifugation and resuspension in PBS, counted using a hemocytometer, then the appropriate volume of yeast suspension was added to BMM. On day 0, BMDMs were infected by replacing the media with 0.5 ml yeast suspension in BMM, with a total of 1 x 10^5^ yeasts/well (MOI = 1). Media was replaced with 0.5 ml fresh BMM for wells containing uninfected BMDMs. Uninfected BMDMs were lysed on day 0 by removing the media and replacing it with 0.5 ml 0.2% Tween-20 in distilled water, and the lysate was collected and stored in 1.5 ml microcentrifuge tubes at 4°C. The BMDMs were fed on day 2 by adding 200 μl BMM. On days 0, 1, 3, 5 and 7, spent media (175 μl) was collected from wells in and stored in 1.5 ml microcentrifuge tubes at 4°C for later analysis in the LDH assay.

### Imaging of Es. africanus infected BMDMS

On imaging days, the media was removed and replaced with 250 μl pre-warmed 4% PFA for 30 min. The PFA was then removed and replaced with PBS. The slides were imaged using a Zeiss Axiovert 200M inverted fluorescence microscope with a Zeiss AxioCam HSc camera.

### Immunofluorescence of FLAG-Pb03 Cbp1 during macrophage infection

1.5x10^5^ BMDMs were plated on 12 mm circular coverslips (Fisher Scientific 12-545-80) in a 24-well plate and infected as described above with the following *Hc* strains at an MOI of 2; *cbp1* mutant with wildtype G217B Cbp1 and 3XFLAG-Pb03 Cbp1. At 24 and 48 hours post infection, cells were fixed with 4% paraformaldehyde for 15 minutes at room temperature and washed 3 times with d-PBS to remove residual paraformaldehyde. Fixed cells were permeabilized with 0.25% Triton-X-100 in PBS for 10 min and then washed 3 times with PBS. Nonspecific binding sites were blocked by incubation with blocking solution (10% goat serum, 22.52 mg/mL glycine, in 0.1% PBST (PBS +0.1% Tween20)) for 30 min at room temperature and then washed 3 times with PBST. To remove non-specific binding to any other mouse IgG Fc receptors commonly found in macrophages, cells were incubated with Goat anti-Mouse F(ab) fragment (abcam ab6668) at 0.1 mg/mL in blocking solution for 1 hour at room temperature. Cells were then incubated with a monoclonal primary mouse anti-FLAG M2 antibody (Millipore Sigma F3165) at a dilution of 1:200 in blocking solution and incubated in a light-blocking incubation chamber overnight with 4°C. Coverslips were washed 3 times with block for 5 min to wash off any remaining primary antibody. Coverslips were incubated with a polyclonal **I**gG (H+L) Highly Cross-Adsorbed Goat anti-Mouse, Alexa Fluor 488 (Fisher Scientific A11029) secondary at a dilution of 1:500 in blocking solution for 1 hour at room temperature in a light-blocking incubation chamber. Coverslips were then washed 3 times with PBST for 5 minutes and then briefly rinsed in double distilled water and the excess liquid was absorbed by the corner of a kimwipe. Coverslips were mounted on glass slides with a drop of Vectashield Mounting media with DAPI and 1:100 Calcofluor White (1 mg/mL). Coverslips were sealed to the glass slide with clear nail polish. Images were taken on a Nikon CSU-X1 spinning disk confocal microscope.

### Affinity purification of 1x/2xstrep- tagged secreted proteins

1xstrep/2xstrep-tagged proteins (2xstrep-eGFP, 1xstrep G217B *Hc* Cbp1, 2xstrep *Pb03* Cbp1, and Yps-3-1x/2xstrep) were purified from either *Hc* culture supernatants or from *Hc*-infected macrophage lysates. In the case of *Hc* culture supernatants, 25 mL of *Hc* culture was grown for 4 days at 37°C with 5% CO_2_ and passed through a 0.22 μm syringe filter. Roche cOmplete Mini Protease Inhibitor Cocktail tablet (Roche 04693124001) was added to the filtered supernatant which was then concentrated using 3000 MWCO Amicon 15 mL centrifugal filters (Millipore Sigma UFC900324) to approximately 500 μL and then diluted back to 1 mL total with d-PBS. To generate *Hc*-infected macrophage lysates, 4 plates of BMDMs seeded at a density of 2.25x10^7^ were infected with *Hc* at an MOI = 5. At 24 hours post infection, the adherent cells were scraped in 10 mL of cold d-PBS and pelleted at 1500 rpm for 10 min at 4°C. After decanting the d-PBS, the cell pellet was suspended in 1 mL of cold lysis buffer (50 mM TRIS-HCl pH 7.5, 150 mM NaCl, 1 mM EDTA, 0.5% NP-40, 1 tablet of Roche cOmplete Protease inhibitor Cocktail, and 1 tablet of Millipore Sigma PhosStop (4906845001). The lysate was sonicated 2 times for 5 seconds at setting 2 using a Fisher Scientific Sonic Dismembrator Model 100 with 1 min on ice in between. The cells were further lysed for 1 hr at 4°C with a rotisserie revolver and the lysate was clarified with a 20 min spin at 13,000 rpm at 4°C. Both the *Hc* supernatant and macrophage lysates were applied to pre-washed Magstrep “type3” XT beads (iba Life Sciences 2-4090-002) and allowed to bind overnight at 4°C on a rotisserie revolver. The Magstrep beads with bound strep-tagged proteins were then washed three times with wash buffer Buffer W (iba Lifesciences 2-1003-100) and eluted with Buffer BXT (iba Lifesciences 2-1042-025).

### CCF4-AM assay

The functionality of strep-Beta-Lactamase expressed by *Hc was* determined by using concentrated culture supernatants with 0.25 mg/mL of Nitrocefin (Toku-E N005) and incubated for 30 minutes at room temperature and the colorimetric change was monitored.

For the CCF4-AM assay, 1x10^7^ BMDMs were plated in non-TC treated 10 cm plates and infected with the following strains at an MOI of 2 for 24 hours; “WT” G217B *ura5*△ with the URA5 plasmid, G217B *ura5*△ with an episomally expressed strep-Beta Lactamase driven by the Cbp1 promoter. BMDMs were also spin infected with the following *Legionella pneumophila* strains at an MOI of 100 for 4 hours prior to the CCF4-AM assay; “WT” flaA, BlaM-RalF, and “dotA” dotA, BlaM-RalF. Cells were scraped and washed with cold Flow Buffer (PBS with 2% FBS, 2 mM EDTA) with Probenecid (Life Technologies P36400). Washed cells were incubated with the LiveBLAzer FRET-B/G Loading Kit with CCF4-AM dye (Life Technologies K1095) for 90 minutes at room temperature in a light protected environment. Dyed cells were analyzed by flow cytometry on FACSAria Fusion “Jabba the Hutt” in UCSF Parnassus Flow Core with the following lasers; AmCyan (515/20 VioletE), PacBlu (450/50 Violet F), and FITC (515/20 Blue B). Data was analyzed with FlowJo V10.7.

### Yps3-6xHis purification

3 Liters of G217B *Hc* expressing Yps3-6xHis were grown at 37°C with 5% CO_2_ for 5 days. The supernatant was sterile filtered through a 0.22 μm 0.5 or 1L vacuum filter and concentrated using the Amicon 200 mL Stirred Cell (UFSC20001) with a 3 kDa NMW Ultrafiltration Disc (Millipore PLBC06210) and Amicon Ultra 15 mL Centrifugal Filters with a 3kDa NMW (Millipore UFC9003). The concentrated supernatant was applied to a cobalt resin to achieve a His-tag pulldown using the HisPur Cobalt Purification Kit (ThermoScientific 90092). The elutions were then applied to a cation exchange column to purify Yps3-6xhis. The majority of the Yps-3-6xhis emerged with Cbp1 in the flow-through.

### Yps3-6xhis and Cbp1 protein interaction assays

Concentrated supernatant was diluted 1:50 in Mono A buffer (20mM Tris-HCl pH 7.0, 20 mM NaCl). The sample was applied to a HitrapS cation exchange column and eluted with 25% MonoB buffer (20 mM Tris-HCL pH 7.0, 1 M NaCl). The flow-through fractions contained both Cbp1 and Yps3-6xHis, as a complex of these proteins would retain a negative change at the buffered pH. The complex was validated by passage through a Superdex75 Size Exclusion column using 50 mM Tris pH 7.0, 150 mM NaCl. Both Cbp1 and Yps-3-6xHis eluted in a monodispersed peak. To determine at what molar ratios this complex of Yps-3 and Cbp1 forms at pure Yps-3-6xHis and G217B Cbp1, were mixed in different molar ratios and pulled down with NiNTA resin. For the Yps3-6xhis pulldown, the SEC flowthrough that contained both Cbp1 and Yps3-6xhis was incubated with 20 uL of washed 50% NiNTA bead slurry for 30 minutes at room temperature with shaking and then the beads were washed with 20 column volumes of NiNTA wash buffer (50 mM Tris-HCl pH 7, 150 mM NaCl, and 30 mM of Imidazole). The complex was eluted with 6 column volumes of NiNTA elution buffer (50 mM Tris-HCl pH 7, 150 mM NaCl, 500 mM of Imidazole). We verified the identity of the proteins in the complex by western blot using native antibody for Cbp1 and a mouse anti-his tag antibody (ABclonal AE003) for Yps3-6xhis.

### RNA isolation and RT-PCR

For RNA isolation from cultured cells, BMDMs were seeded (1x10^6^ cells per well of a 6-well plate) and infected in triplicate as described. Triplicate wells of infected macrophages were lysed in 1mL total of QIAzol (Qiagen). Total RNA was isolated from the aqueous phase using Econo-spin columns (Epoch Life Science) and then subjected to on-column PureLink DNase (Invitrogen) digestion. To generate cDNA, 2–4 μg total RNA was reverse transcribed using Maxima Reverse Transcriptase (Thermo Scientific), an oligo-dT primer, and pdN9 primers following manufacturer’s instructions. Quantitative PCR was performed on 1:10 to 1:50 dilutions of cDNA template using FastStart SYBR Green MasterMix with Rox (Roche). Reactions were run on an Mx3000P machine (Stratagene) and analyzed using MxPro software (Stratagene). Cycling parameters were as follows: 95°C for 10 min, followed by 40 cycles of 95°C (30 s), 55°C (60 s), and 72°C (30 s), followed by dissociation curve analysis. Abundances of CHOP and TRIB3 were normalized to HPRT levels.

### Mass spectrometric analyses

Mass spectrometry was performed by the Vincent J.Coates Proteomics/Mass Spectrometry Laboratory at UC Berkeley. For 2D “mudPIT” samples, a nano LC column was packed in a 100 μm inner diameter glass capillary with an emitter tip. The column consisted of 10 cm of Polaris c18 5 μm packing material (Varian), followed by 4 cm of Partisphere 5 SCX (Whatman). The column was loaded by use of a pressure bomb and washed extensively with buffer A (see below). The column was then directly coupled to an electrospray ionization source mounted on a Thermo-Fisher LTQ XL linear ion trap mass spectrometer. An Agilent 1200 HPLC equipped with a split line so as to deliver a flow rate of 300 nl/min was used for chromatography. Peptides were eluted using an 8-step MudPIT procedure [67]. Buffer A was 5% acetonitrile/ 0.02% heptaflurobutyric acid (HBFA); buffer B was 80% acetonitrile/ 0.02% HBFA. Buffer C was 250 mM ammonium acetate/ 5% acetonitrile/ 0.02% HBFA; buffer D was same as buffer C, but with 500 mM ammonium acetate. For simple 1D samples the column consisted of 10 cm of Polaris c18 only, and was eluted with a linear gradient to 60% buffer B over 90 min.

Protein identification and quantification were done with IntegratedProteomics Pipeline (IP2, Integrated Proteomics Applications, Inc. San Diego, CA) using ProLuCID/Sequest, DTASelect2 and Census [68–71]. Tandem mass spectra were extracted into ms1 and ms2 files from raw files using RawExtractor [72]. Data was searched against combined *H*. *capsulatum* and mouse databases supplemented with engineered sequences as needed. This combined database was further supplemented with sequences of common contaminants and concatenated to a decoy database in which the sequence for each entry in the original database was reversed [73]. LTQ data was searched with 3000.0 milli-amu precursor tolerance and the fragment ions were restricted to a 600.0 ppm tolerance. All searches were parallelized and searched on the VJC proteomics cluster. Search space included all fully tryptic peptide candidates with no missed cleavage restrictions. Carbamidomethylation (+57.02146) of cysteine was considered a static modification. We required 1 peptide per protein and both trypitic termini for each peptide identification. The ProLuCID search results were assembled and filtered using the DTASelect program [69, 70] with a peptide false discovery rate (FDR) of 0.001 for single peptides and a peptide FDR of 0.005 for additional peptide s for the same protein. Under such filtering conditions, the estimated false discovery rate was about 1% for the datasets used.

### SDS-PAGE Protein gel and western blot analysis

Protein samples were mixed with 4X Protein Loading Buffer (Li-COR 926–31097) and 1:20 DTT (1M DTT) and denatured at 95°C for 5 minutes. Proteins were separated by SDS-PAGE on NOVEX-NuPAGE 4–12% BIS-TRIS gels with MES Running Buffer and Precision Plus Dual Xtra Protein Standards (Bio-Rad161-0377) were used to estimate the molecular weight of proteins. For Western blots, the SDS-PAGE separated proteins were transferred to nitrocellulose membranes. Non-specific binding to the membrane was blocked with Odyssey PBS Blocking Buffer (Fisher Scientific NC9877369) and probed with the antibodies listed below. Blots were imaged on an Odyssey CLx and analyzed using ImageStudio2.1 (Li-COR). The following primary antibodies were used: monoclonal mouse anti-FLAG M2 (Millipore Sigma F3165), monoclonal mouse anti-strep (StrepMab) Classic iba LifeSciences 2-1507-001), custom generated Rabbit anti-Cbp1 (Rb83), custom generated Rabbit anti-RYP1, mouse anti-LAMP1 (cell signaling D401S), mouse anti-alpha-tubulin (Novus biological DM1A), Rabbit anti-calnexin (abcam 22595), mouse anti-His (ABclonal AE003).

### In vitro Histoplasma capsulatum growth

Two-day, late-log cultures of the G217B *ura5Δ*, *yps-3Δ* strain carrying the *URA5* plasmid and the *yps-3Δ* + *YPS-3* complemented strain were used to inoculate 50 mL of HMM medium to a starting OD_600_ = 0.05 in triplicate. At each timepoint, samples were removed from each culture, mixed well, diluted to be within the linear range, and analyzed to determine their OD_600_.

### Mouse infections

Eight-to-twelve week-old female C57Bl/6 mice (Jackson Laboratory stock 000664) were anesthetized with isoflurane and infected intranasally with wild-type *H*. *capsulatum* (G217B *ura5Δ* + *URA5*), the *yps-3* mutant (*yps-3Δ* + *URA5*) or the complemented stain (*yps-3Δ* + *YPS-3*). The inoculum was prepared by collecting mid-logarithmic phase (OD_600_ = 5–7) yeast cultures, washing once with PBS, sonicating for 3 seconds on setting 2 using a Fisher Scientific Sonic Dismembrator Model 100, counting with a hemacytometer, and diluting with PBS so the final inoculum was approximately 25 μL. To monitor survival, animals were infected intranasally with 1.0 × 10^6^ yeast per mouse. Infected mice were monitored daily for symptoms of disease, including weight loss, hunching, panting, and lack of grooming. For survival curve analysis, mice were euthanized after they exhibited 3 days of sustained weight loss greater than 25% of their maximum weight in addition to one other symptom. To confirm correct inoculum and proper intranasal infection, the inoculum itself and the 4 hr post-infection lungs for 2 mice per condition were harvested and homogenized in PBS and plated on brain heart infusion agar plates supplemented with 10% sheep’s blood. CFU’s were enumerated after 10–12 days of growth at 30°C.

## Supporting information

S1 Fig InstantBlue stained SDS-PAGE gel of Cbp1 homologs purified directly from culture supernatants. Cbp1 was purified from supernatants of the indicated strains (*Hc* G217B; *Hc* G186AR; *Hc* H88; *Hc* G217B *ura5 cbp1* carrying either Pb03, *E*. *crescens*, or *Es*. *africanus* Cbp1). Purified proteins were subjected to SDS-PAGE and InstantBlue staining.
(EPS)

S2 Fig Comparisons of the previously published G186AR NMR structure with the H88 and Pb03 Cbp1 crystal structure. A. Highlight of the aromatic (colored mint green) and aliphatic (colored red) residues to showcase the greasy patch formed by the C-terminal helices of each monomer and the aromatic residues packing into the hydrophobic core. B. Alignment of the Pb03 and H88 crystal structures with the G186AR NMR structure shows major differences in the arrangement of the helices. The RMSD scores from the alignments of G186AR to Pb03 and H88 are 5.608 and 5.879 respectively. C. Charge distribution over the surface of the protein structures shows major differences in the negative charge at neutral pH. Negative charge is indicated on surface with red, positive charge with blue, and neutral with white. The Pb03 dimer has a negative charge patch at the bottom of the C-terminal helical bundles, whereas the H88 dimer is only partially negative there. The H88 dimer has a negative groove that runs along the side of the protein. Only the Pb03 structure has a partial positive charge over the N-terminal helices. The electrostatic surfaces were determine using the APBS/PDB2PQR software (48, 49). D. The tertiary structures of *Emergomyces homologs* were modelled on the Pb03 Cbp1 backbone using MODELLER [47] and the surface charge distribution was determined using the APBS/PDB2PQR software.
(TIF)

S3 Fig Generation of chimeric *Hc-E*. *crescens* and Pb03-*E*. *crescens* alleles. A. Illustration of residue changes made to *E*. *crescens* Cbp1 to generate chimeras with either G217B or Pb03 Cbp1. The protein alignments were compared to the alanine scan of G217B to identify residues that are required for lysis in G217B Cbp1 but not conserved in *E*. *crescens* Cbp1. Based on the H88 and Pb03 Cbp1 structures, we determined which of these residues were oriented outwards on the surface. These residues were also checked for whether their side chains differed in size, polarity, or charge. The proposed chimeric constructs are illustrated. The green (G217B) and yellow (Pb03) residues represent those in the N-terminal helix whereas the darker blue (G217B) and lighter blue (Pb03) residues represent those in the C-terminal loops. B. The selected differential residues that were mutated in the N-terminal helix and C-terminal loops are highlighted in green and teal respectively in the Pb03 and H88 structures.
(EPS)

S4 Fig Controls for fractionation to assess rupture of small membranous compartments or *Hc*-containing phagosome. A. BMDMs were mock-infected (uninf) or infected with either the “WT” strain (*Hc* G217B *ura5*^*-*^ carrying a *URA5* vector control), the *cbp1* mutant (*Hc* G217B *ura5*^*-*^ *cbp1*^*-*^) carrying either the vector control or G217B Cbp1 with 3XFLAG (G217B Cbp1 3XFLAG), Pb03 Cbp1 with 3XFLAG (Pb03 Cbp1 3XFLAG), WT G217B *ura5*^*-*^ carrying Yps-3 3XFLAG (Yps-3 3XFLAG), or the *Hc* G217B *ura5*^*-*^ *yps3*^*-*^ mutant (*yps3*^*-*^). Macrophage lysates were subjected to fractionation to separate cytosolic and membrane fractions. The fraction containing *Hc* yeast and macrophage nuclei with some contaminating cytosolic and membrane components was analyzed by SDS-PAGE and Western blotting using anti-Calnexin (endoplasmic reticulum), anti-α-Tubulin (cytosol), anti-FLAG or anti-Cbp1 antibodies. B. The cytosolic, membrane, and *Hc*/nuclear fractions were subjected to SDS-PAGE and Western blotting using anti-LAMP1 (marking the late endosomes and lysosomes) or anti-Ryp1 (an *Hc* transcription factor that is not released from the *Hc* cells, indicating that they are intact). C. Concentrated culture supernatants from G217B *Hc* ura5△ isolates expressing strep-tagged β-Lactamase were subjected to SDS-PAGE and Western blot analysis using an anti-strep antibody to monitor for robust secretion of the enzyme (top). The same culture supernatants were combined with Nitrocefin to monitor for a color change from yellow to red to indicate β-Lactamase activity. The isolates with the highest β-Lactamase activity were used for the CCF4-AM assay in Fig 5.
(EPS)

S5 Fig BMDM infections with *Hc* strains expressing tagged versions of *Hc* or Pb03 Cbp1. A. BMDMs were mock-infected (uninf) or infected with the “WT” strain of *Hc* G217B *ura5*^*-*^ carrying the vector control or the *Hc* G217B *cbp1* mutant strain carrying either the *URA5* vector control or 3 independent transformants of Pb03 Cbp1 tagged with 3XFLAG. LDH release was monitored over time. B. BMDMs were mock-infected (uninf) or infected with the “WT” strain of *Hc* G217B *ura5*^*-*^ carrying the *URA5* vector control or the *Hc* G217B *cbp1* mutant strain carrying either the *URA5* vector control, untagged G217B Cbp1, or 3 independent transformants of G217B Cbp1 tagged with 3XFLAG. LDH release was monitored over time. C. BMDMs were mock-infected (uninf) or infected with the “WT” strain of *Hc* G217B *ura5*^*-*^ carrying the vector control or with the *Hc* G217B *cbp1* mutant carrying G217B Cbp1 or Pb03 Cbp1 untagged or tagged with 1XStrep or 2XStrep respectively. Other strains included *Hc* G217B *cbp1* mutant transformed with a variety of *Emergomyces* Cbp1 alleles. LDH release was monitored over time.
(EPS)

S6 Fig Determination of the optimal molar ratio for Cbp1:Yps-3 complex. Purified Cbp1:Yps-3-6xhis were mixed in the following molar ratios: 1:1, 1:2, 2:1, and 5:1 and then Yps-3-6xhis was pulled down with Ni-NTA beads to determine if it co-purified with Cbp1.
(EPS)

S7 Fig Generation and characterization of the *yps-3* deletion mutant in *Hc* G217B using CRISPR-Cas9. A. Schematic of CRISPR sgRNA guide target sites (triangles) for the *YPS-3* locus and the location of internal and external primers that were used to probe for the deletion of the locus in panel B. Purple box indicates the *YPS-3* coding sequence. B. PCR of *YPS-3* locus from genomic DNA from WT *Hc* G217B and *yps-3*Δ mutant utilizing the external or internal primers designated in A. C. *In vitro* growth curve analysis of G217B *ura5△* (WT), the G217B *ura5△ yps-3△* (*yps-3△*) both with the *URA5* plasmid, and the *yps-3△* carrying the complementation vector (*yps-3△*+*YPS-3*). Yeast cells were inoculated in HMM starting at an OD = 0.05 and the subsequent OD_600_ was monitored over time. The average of three measurements ± standard deviation is shown. D. TRIB3 transcript levels relative to the housekeeping gene mHPRT were assessed by qRT-PCR at the indicated timepoints for uninfected, Tunicamycin-treated, or BMDMs infected with WT, *cbp1△*, *yps-3△*, and *yps-3△*+*YPS-3*. Asterisks represent p-value < 0.05 by Student’s t-test.
(EPS)

S1 Table Mass spectrometry analysis of Cbp1 peptides. Peptide data for mass spectrometric analysis of Cbp1 is shown for Cbp1 proteins from G217B, G186AR, H88, *Pb03*, *Es*. *africans*, *E*. *crescens*, *Es*. *orientalis*, *Es*. *pasteurianus*, and the Cbp1 hybrids generated from *E*. *crescens–*G217B chimeras and *E*. *crescens–Pb03* chimeras.
(XLSX)
