# Supplementary figures and images for: Cbp1, a fungal virulence factor under positive selection, forms an effector complex that drives macrophage lysis
Source: PLoS Pathog. 2022 Jun 22;18(6):e1010417. doi: 10.1371/journal.ppat.1010417 (PMC9255746; doi:10.1371/journal.ppat.1010417)

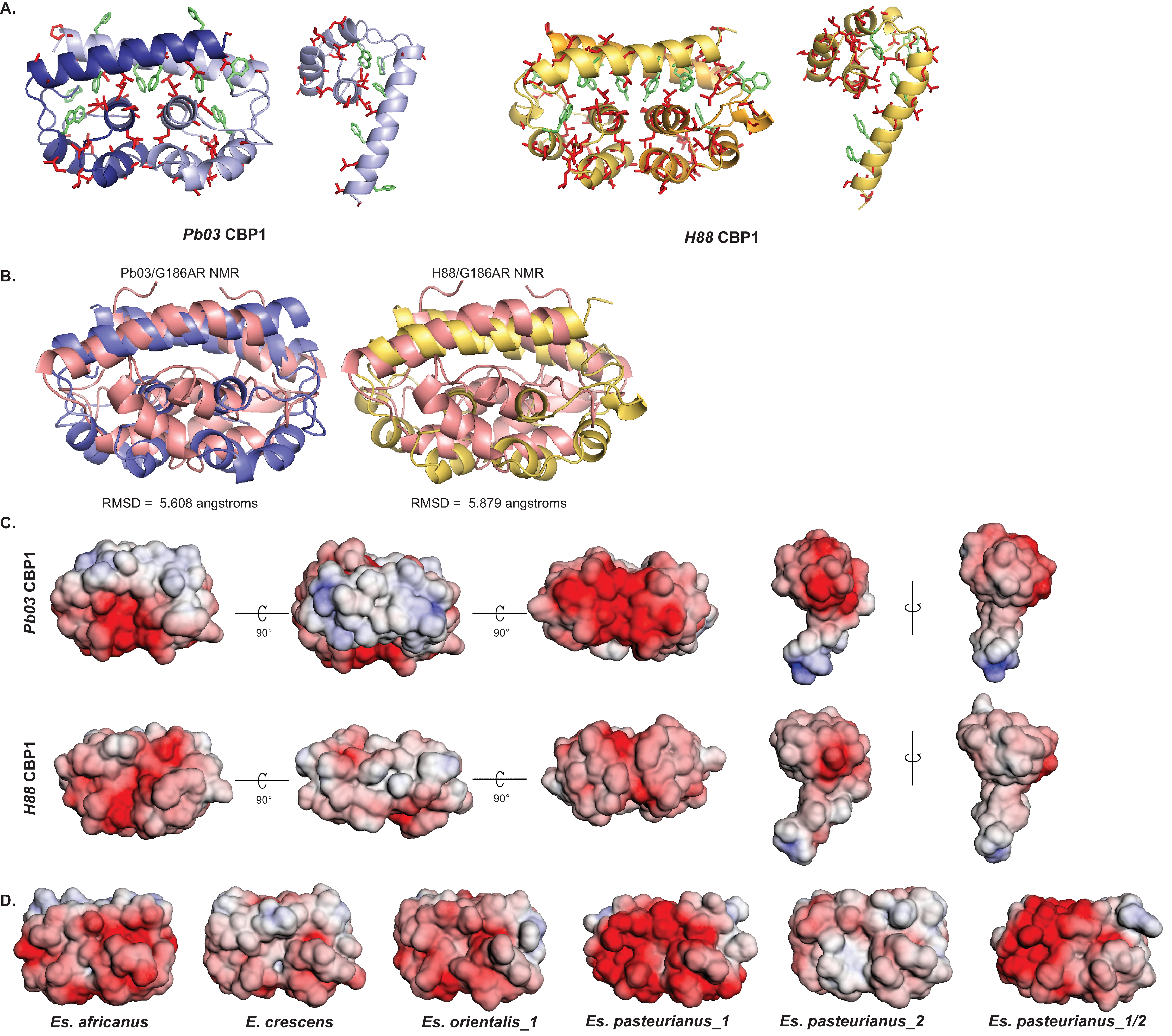

Supplement: S2 Fig — A. Highlight of the aromatic (colored mint green) and aliphatic (colored red) residues to showcase the greasy patch formed by the C-terminal helices of each monomer and the aromatic residues packing into the hydrophobic core. B. Alignment of the Pb03 and H88 crystal structures with the G186AR NMR structure shows major differences in the arrangement of the helices. The RMSD scores from the alignments of G186AR to Pb03 and H88 are 5.608 and 5.879 respectively. C. Charge distribution over the surface of the protein structures shows major differences in the negative charge at neutral pH. Negative charge is indicated on surface with red, positive charge with blue, and neutral with white. The Pb03 dimer has a negative charge patch at the bottom of the C-terminal helical bundles, whereas the H88 dimer is only partially negative there. The H88 dimer has a negative groove that runs along the side of the protein. Only the Pb03 structure has a partial positive charge over the N-terminal helices. The electrostatic surfaces were determine using the APBS/PDB2PQR software (48, 49). D. The tertiary structures of Emergomyces homologs were modelled on the Pb03 Cbp1 backbone using MODELLER [47] and the surface charge distribution was determined using the APBS/PDB2PQR software. (TIF) [file ppat.1010417.s002.tif]
